# Changes in child mortality and population health following 10 years of health systems strengthening in rural Madagascar: A longitudinal cohort study

**DOI:** 10.1371/journal.pmed.1004549

**Published:** 2025-10-07

**Authors:** Andres Garchitorena, Ann C. Miller, Hobisoa L. Razanadranaivo, Luc Rakotonirina, Sarah-Anne Barriault, Benedicte Razafinjato, Jacques Aubin Kotchofa, Feno Rafenoarimalala, Rado J. L. Rakotonanahary, Felana A. Ihantamalala, Michelle V. Evans, Andoniaina Tojoharimanda Tolojananahary, Vero Ramanandraitsiory, Andriatiana Tsitinomen’nyaina, Fiainamirindra Anjaratiana Ralaivavikoa, Estelle M. Raza-Fanomezanjanahary, Marius Randriamanambintsoa, Samuel Andrianambinina, Lethicia Lydia Yasmine, Megan B. Murray, Michael L. Rich, Laura F. Cordier, Karen E. Finnegan, Matthew H. Bonds, Zely Arivelo Randriamanantany

**Affiliations:** 1 MIVEGEC, University of Montpellier, CNRS, IRD, Montpellier, France; 2 NGO PIVOT, Ranomafana, Madagascar; 3 Department of Global Health and Social Medicine, Harvard Medical School, Boston, Massachusetts, United States of America; 4 Direction de la Démographie et des Statistiques Sociales, Institut National de la Statistique, Antananarivo, Madagascar; 5 Ministry of Public Health in Madagascar, Antananarivo, Madagascar; Washington University In St Louis: Washington University in St Louis, UNITED STATES OF AMERICA

## Abstract

**Background:**

Reducing child mortality rates is a unifying goal of the global health and international development communities. In Africa, unambiguous empirical evidence on how health system interventions can drive such reductions has been elusive. This gap in the literature is due to challenges in implementing system-level changes on a scale and pace to have measurable impacts on mortality, and the challenges of collecting adequate data on the population and programs over sufficient time with plausible counterfactuals. This study aimed to assess the population health impact of the first decade of implementation of a health system strengthening (HSS) intervention in a rural district of Madagascar.

**Methods and findings:**

The study is a prospective quasi-experiment using a district-representative cohort of over 1,500 households (five waves of survey collection), in combination with patient data collected across different levels of care (community health workers and health facilities), geographic information systems, and programmatic data to assess changes in mortality, healthcare coverage and utilization from 2014 to 2023. The HSS intervention integrates support to clinical programs with strengthened health system building blocks and social protection at all levels of care of a district health system (community health, primary care centers, and hospital). Under-five, infant and neonatal mortality were estimated at the population level using the synthetic life-table method for DHS surveys. Impact of the HSS intervention on healthcare coverage and utilization was assessed through interrupted time-series analyses. Changes in geographic and financial inequalities in coverage indicators were studied via the relative concentration index and slope index of inequality. Our results show that trends in child mortality rates (neonatal, infant, under-five) decreased in the initial HSS intervention area from 2014 to 2023, but increased in the comparison area as well as the rest of the country over the same period. The HSS intervention was associated with statistically significant increases in service coverage and primary care utilization for a wide range of maternal and child health indicators, as well as reductions in geographic and financial barriers to care. The main limitations of this study were that the intervention was not randomized, and that changes in child mortality were estimated from 5-year averages from repeated cross sections, with overlapping time windows that prevented formal integration into the statistical modeling framework used for coverage indicators.

**Conclusions:**

By measuring both indirect and direct impacts of HSS on population health in a context where health and economic indicators are not otherwise improving, these results provide converging evidence on how strengthening health systems, from community health to hospitals, in low-resource settings increases overall utilization of services, reduces inequities in access to those services, and corresponds with reductions in mortality.

## Author summary

- Why was this study done?
- In sub-Saharan Africa, there is limited prospective evidence on how integrated health system strengthening (HSS) interventions affect child mortality at the population level.
- Most previous studies have focused on discrete interventions or short timeframes, failing to capture broader systems-level impacts on health outcomes and equity.
- This study aimed to evaluate the 10-year impact of a district-wide HSS initiative on child mortality, health service coverage, and inequalities in rural Madagascar.
- What did the researchers do and find?
- We used a quasi-experimental design with a longitudinal cohort of over 1,500 households, health system data, and geospatial analyses from 2014 to 2023.
- The HSS intervention integrated support for clinical services, health system building blocks, and social protection across all levels of care in a district health system.
- Results showed declines in neonatal, infant, and under-five mortality in the HSS catchment, concurrent with increased service coverage, utilization, and reduced geographic and financial inequalities—contrasting with worsening trends elsewhere.
- What do these findings mean?
- Integrated health systems strengthening—across levels of care and health system building blocks—can lead to measurable reductions in child mortality.
- System-wide HSS can simultaneously improve healthcare utilization and reduce disparities by increasing access for poor and remote populations.
- These findings underscore the importance of long-term, integrated HSS strategies for achieving universal health coverage in low-resource settings.
- The main limitations of this study are that the intervention was not assigned randomly, and that changes in child deaths were estimated using five-year averages for each survey year, which covered overlapping time periods.

## Abstract 3

In a longitudinal cohort study, Andres Garchitorena and colleagues assess the effects of a health system strengthening intervention in rural Madagascar.

## Introduction

Since the turn of the millennium, the international development agenda to improve health in low- and middle-income countries has expanded significantly in scope. In addition to disease-specific programs, such as for HIV, tuberculosis and malaria, broader priorities have emerged to strengthen health systems and “ensure healthy lives and promote well-being for all at all ages” [1–3]. Sustainable Development Goal 3 (SDG 3) explicitly targets the achievement of universal health coverage (UHC) and an end to all preventable deaths of newborns and children under-five, over half of which occur in Africa [4,5]. Among population health metrics, child mortality rates stand out for their direct alignment with the ultimate goal of saving lives and as useful metrics that are detectable with standardized tools comparable within and across populations over time [6,7]. Many field-based initiatives specifically focus on reducing under-five (U5) mortality because it is considered measurable within a relevant time frame [8–11].

One of the great paradoxes in global health today is that, although indirect evidence overwhelmingly suggests that child mortality rates in Africa should decline with increased access to maternal and child health services, there is very little prospective evidence – based on standardized metrics – that programs that target U5 mortality are effective in reducing it [8]. This contrasts with the strength of the indirect evidence, which convincingly covers many steps of the relevant pathways: (1) most child deaths across sub-Saharan Africa are due to preventable and treatable infectious diseases, such as malaria, diarrheal disease, and respiratory infections among other common child illnesses [4,5,12]; (2) vaccines and medical care are highly effective in preventing and treating these diseases and in reducing mortality among enrolled patients in trial settings [13–16]; and (3) programs that reduce financial or geographic barriers and strengthen health systems expand access to essential maternal and child health services [17–20]. Based on this body of evidence, modelling efforts such as those using the Lives Saved Tool (LiST) have estimated that child mortality rates would decline by 60%–90% with the scale-up of proven technologies and interventions [19,21–24].

Perhaps the strongest empirical support of population-level health effects in Africa comes from a growing number of bottom–up community-based interventions. These generally report positive impacts on coverage (i.e., the share of the target population receiving a core set of health services) for maternal, neonatal, and child health services [25–28], though the results from larger-scale (typically, multilaterally funded) interventions tend to be weaker [8]. Of the small subset of the literature that reports on mortality rates, findings are mixed when compared to secular trends at the national level, and there is little consistency in the identification of counterfactuals necessary for inferring causality [8,28–32]. Few evaluations have had prospectively identified comparison groups, and many coincide with rapid contemporaneous socio-economic and demographic changes, with limited baseline data [31,32]. How should the lack of clear evidence be interpreted? Does it reflect fundamental limitations of healthcare relative to social and environmental factors [33]? Are programs failing to implement quality care? Or, alternatively, are the data inadequate due to short time series, small sample sizes or missing counterfactuals? These are fundamental questions about which interventions work in real-world settings, what metrics are informative, and at what scale.

We present results on population health after the first decade of an initiative to strengthen the local health system in southeastern Madagascar. A partnership between the Madagascar Ministry of Public Health (MoPH), local communities and a field-based non-governmental organization (NGO), established a model district for universal health coverage, focused on implementing and improving national policies. This intervention (Details in “Methods” section and Table 1) is structured around the integration of clinical programs with strengthened health system pillars and social protection across all levels of care within the district (community health, primary care centers, and hospital) [34]. The research is designed as a prospective quasi-experiment [9], relying on multiple data sources with a true baseline in intervention and comparison groups [35]. These data include a population-representative longitudinal cohort with relatively large sample size (approximately 8,000 individuals), health management information systems (HMIS) from community health and public health facilities, NGO program data, and geographic information systems (GIS).

**Table 1:** Summary of health system strengthening (HSS) activities implemented in Ifanadiana district between 2014 and 2023 at each level of care, classified by building block of HSS^1^ affected and including data sources used for the impact evaluation in this study.

Level of care	Ifanadiana district (HSS + Comparison area)	
**District hospital** Total Ifanadiana: 1 hospital	**(1)** Network of 3 ambulances for referrals and emergency care; infrastructure renovations, provision of medical and non-medical equipment, including full laboratory capacity; social support for vulnerable patients **(2)** Staffing of health workers and non-clinical staff above MoPH norms; trainings for medical staff **(3)** Creation of a hospital-based M&E team to follow-up progress of activities; frequent facility readiness surveys **(4)** Supply chain management to reduce stock-outs, management of hospital pharmacy **(5)** Cost of outpatient and inpatient care fully covered for referred patients (district hospital and tertiary care outside Ifanadiana) **(6)** Creation of a joint MoPH-Pivot executive committee for hospital management and transparency; subcommittees for specific projects **Data:** IHOPE cohort	
	**HSS catchment**	**Ifanadiana district (HSS + Comparison area)**
**Health Centers (PHC)** Total Ifanadiana: 15 HSS catchment: 4 (2014–2016) 6 (2017–2018) 15 (2021)	**(1)** Infrastructure renovations, provision of medical and non-medical equipment; implementation of IMCI and malnutrition protocols for every child under 5, support for maternal health services **(2)** Staffing of PHCs above MoPH norms; frequent trainings for medical staff **(3)** Joint MoPH-Pivot training and supervision to improve HMIS data quality **(4)** Supply chain management, training, and reduction of stock-outs **(5)** Essential medicines and consumables provided free of charge to all patients **(6)** Close collaboration with district health managers for the planning and implementation of activities **Data:** Aggregated PHC consultations, patient-level PHC consultations, IHOPE cohort	**(1)** Provision of basic medical and non-medical equipment^2^ **(2)** Staffing to bring all health centers up to MoPH norms; trainings for medical staff **(3)** Additional support to training and supervision and district gatherings **(5)** Basic package of health services free of charge for children under five years and pregnant women^2^ **Data:** Aggregated PHC consultations, IHOPE cohort
**Community Health (CHS)** Total Ifanadiana: 195 fokontany HSS catchment: 21 (2014–2016) 43 (2017–2018) 80 (2019–2020)	**(1)** Construction of 81 community health posts by community, with Pivot support; implementation of IMCI and malnutrition protocols for every child U5 **(2)** Training, coaching and monthly supervision of community health workers by mobile teams of trained nurses and midwives **(3)** Joint MoPH-Pivot training to improve HMIS data quality; support for use of electronic tools for data collection and decision-making **(4)** Monthly provision of MNCH medicine stocks to CHWs and follow-up of medicine stock use **(5)** Cost of MNCH medicine stocks fully covered by Pivot; financial incentives to CHWs for stock management and attendance to supervisions **(6)** Community engagement and participation **Data:** Aggregated PHC consultations, IHOPE cohort	**(1)** Provision of non-medical equipment and supplies **(2)** Training; monthly performance evaluation at health centers; on-site coaching by technical assistants every two months^3^ **(4)** Provision of a free initial stock of products and medicines (subsequent stocks are purchased by CHWs)^3^ **(5)** CHWs make a profit from a small margin in the sale of medicines (except those in HSS catchment) **Data:** IHOPE cohort

1 Building blocks of HSS: **(1)** Service delivery **(2)** Health workforce **(3)** Health information systems **(4)** Medicines and supplies **(5)** Financing **(6)** Leadership and governance.

2 Implemented by PAUSENS program (World Bank).

3 Implemented by Mikolo and ACCESS programs (USAID).

More information about the HSS intervention is available in S1 Table.

Previously published results from this setting during the early years of the health system strengthening (HSS) initiative (2014–2018) demonstrated rapid increases in the utilization of primary and secondary care [36,37], in the coverage of maternal, neonatal, and child health services [27,38], and in measures of the quality of care provided by the public health system [39]. However, similar to much of the reported literature, those measured improvements in health system performance did not coincide with reductions in mortality rates [27,38]. Subsequent geographic analyses showed that despite the increases in overall service coverage, stark and persistent geographic barriers to the health system reinforced inequalities in healthcare access [37,40–42]. Those insights led to renewed investments in community health programs and other efforts to reduce geographic barriers such as patient referral programs [34]. Now, with longer time series, more detailed geolocated patient data, and with the benefit of frequent household surveys from a single cohort (rather than repeated cross sections) in a country where general health indicators are not otherwise improving, this study aimed to evaluate whether and how an adaptive health system strengthening intervention can lead to improvements in access to higher quality services, reductions in inequality in access, and corresponding decreases in child mortality.

## Methods

### Study aims and design

The goal of this study was to evaluate the effectiveness of a local HSS initiative in improving population health in the rural district of Ifanadiana, Madagascar. Specific aims were to: (1) estimate changes in child mortality rates (U5, infant, neonatal) in the intervention and non-intervention areas between 2014 and 2023, as HSS activities were rolled out across the district; (2) evaluate the impact of the HSS intervention on healthcare utilization and care-seeking behaviors over time; and (3) measure impacts on economic and geographic inequalities in access to care over time. Our main hypothesis was that the HSS initiative would lead to reductions in mortality rates, increases in service coverage and utilization, and reductions in inequalities in access to care. For this, we used a quasi-experimental design that includes adjacent intervention and comparison (non-intervention) areas within the same district. The outcomes were pre-specified at baseline of the intervention [35], and the initial households included in the district-representative baseline survey were subsequently enrolled in an open prospective cohort study [9]. At baseline, there were no statistically significant differences in health system utilization or service coverage [35]. Over time, similar to a stepped-wedge trial but without randomization, the programs expanded and improved across a gradually widening intervention area until the entire district was covered by the HSS intervention.

The most fundamental outcome of interest is child mortality. However, the prospective cohort was established at the household level, which did not allow for individual child follow-up or clean statistical testing. Instead, five-year birth histories were analyzed from repeated cross-sections in the cohort using standard methods for demographic and health surveys (DHS). This enabled comparison of trends at the national and international level but resulted in many mortality observations that included periods both exposed and unexposed to the intervention. We present trends in child mortality in the intervention areas, the rest of the district (comparison areas), and the rest of Madagascar. To understand the potential role of health system interventions in explaining these trends, we analyze changes in health system performance over the same time period in the intervention and comparison areas. This included programs focused on the provision of health services (clinical programs and HSS pillars) and those focused on increasing access to those services (user-fee removal). We first analyze impacts on health system utilization from high resolution (but not population-representative) health facility data (i.e., consultations from the HMIS). We assess program impacts on service coverage outcomes using population-representative household surveys in the cohort. This is followed by an analysis of impacts on equity in access, with a particular focus on specific health program effects on geographic barriers to care. Details of the methods are described below. This study is reported as per the Strengthening the Reporting of Observational Studies in Epidemiology (STROBE) guideline (S1 Checklist).

### Study site and HSS intervention

Ifanadiana is a rural district of approximately 200,000 people located in the region of Vatovavy, in Southeastern Madagascar. Per MoPH norms, Ifanadiana district has one reference hospital, one main primary care health center (PHC2) for each of its 15 communes (subdivisions of a district with approximately 15,000 people – two of which were created in 2016 and 2018), six additional basic health centers (PHC1) for its larger communes (larger communes have both a PHC2 and a PHC1), and one community health site (CHS) with two community health workers (CHWs) for each of its 195 fokontany (subdivision of a commune with approximately 1,000 population). The health system strengthening intervention is implemented by the NGO Pivot across all three levels of care in the district (community, health center, and hospital) and is structured around the integration of clinical programs with strengthened health system pillars and social protection. The clinical programs include child health with a focus on malnutrition and integrated management of childhood illness (IMCI), maternal and reproductive health services, and infectious disease programs targeting tuberculosis, malaria and emerging diseases. Strengthening of the health system pillars focuses on improving “readiness”, including infrastructure, trained staff, equipment, procurement systems, and an ambulance network. User fees are covered by the NGO and additional social support is provided to vulnerable patients to offset indirect costs of care. A summary of the intervention is available in Table 1, with further details in S1 Table.

**Fig 1:**
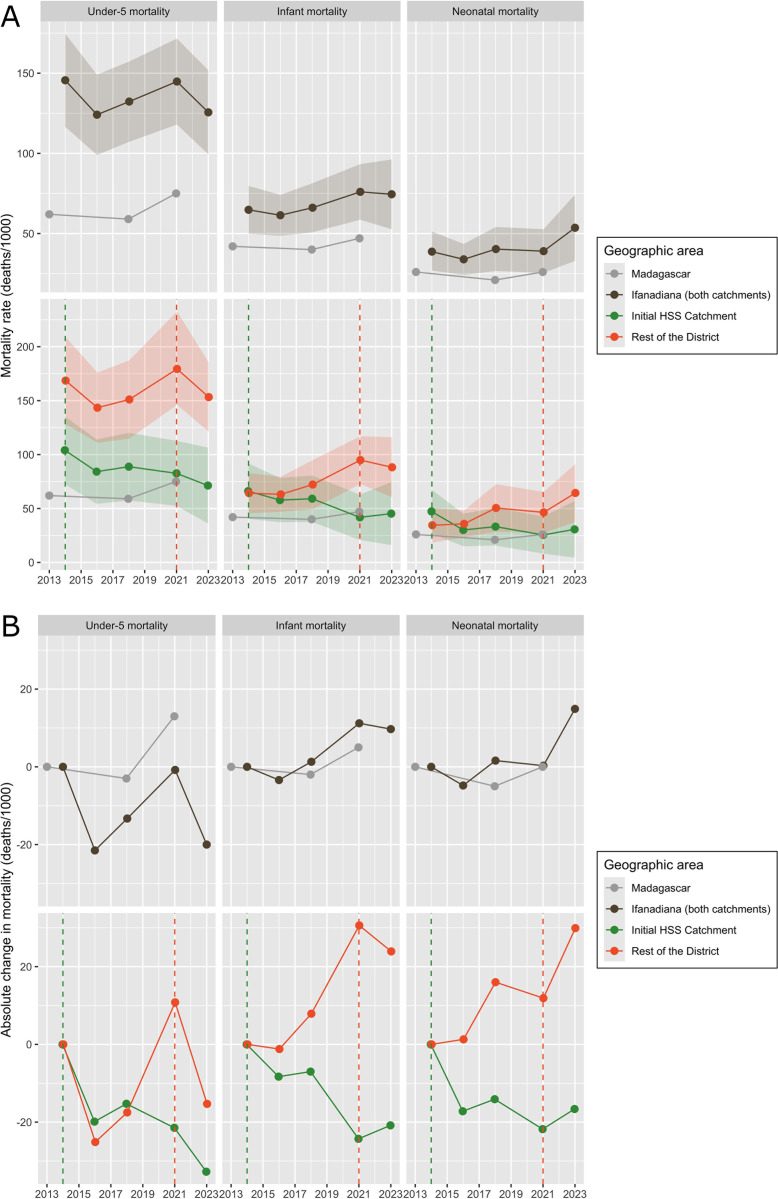
Under-five, infant and neonatal mortality improved in intervention areas while outcomes in comparison areas (as well as the country as a whole) generally worsened over the study period, 2013–2023. **(A)** Each dot represents the average mortality rate in the 5 years preceding the survey, together with 95% confidence intervals. **(B)** Each dot represents the difference in mortality between each time point and the 2013–2014 baseline for each geographic area. Estimates for Ifanadiana district and HSS catchments were estimated from the IHOPE cohort. Estimates for Madagascar were obtained from reports of the national surveys conducted by INSTAT in Madagascar during the same period (ENSOMD 2013, MICS 2018, DHS 2021). These surveys do not report 95% confidence intervals of mortality estimates, but they have larger sample sizes than the IHOPE cohort. *Abbreviations: DHS Demographic and Health Survey; ENSOMD Enquête de Suivi des Objectifs du Developpement du Millenaire à Madagascar; HSS Health Systems Strengthening; IHOPE Ifanadiana Health Outcomes and Prosperity longitudinal Evaluation; INSTAT Institut National des Statistiques; MICS Multiple Indicators Cluster Survey.*

The core activities were rolled out in the district in three waves. The first three years (2014–2016) covered four communes with approximately one third of the population of Ifanadiana (referred to as “initial HSS catchment”). In 2017–2020, HSS activities expanded to three additional communes, and then expanded again in 2021, such that by 2021 all PHC2s were supported (PHC1s have not yet been supported). Some activities, such as medical staff recruitments and strengthened information systems spanned the whole district (S1 Table). The HSS intervention, initially modelled after an earlier experience in HSS in Rwanda [31], has been progressively adapted over time in collaboration with the government to respond to coverage gaps and intervention deficiencies identified through an iterative learning process that uses health system and population-level data (S1 Table). The initial HSS intervention area and subsequent waves of expansion across the district were guided by operational considerations by the NGO’s implementation staff in consultation with the MoPH. In particular, implementation teams chose catchment populations where the health facility was accessible by 4 × 4 vehicles to facilitate the first years of operations. As a result, mortality rates were lower at baseline in the intervention area than in the rest of the district (Fig 1), and some socio-economic indicators were more favorable, while others were less so [35].

In addition to the HSS intervention, during this period, the population of Ifanadiana benefited from other notable programs that covered both the intervention and comparison areas. The PAUSENS project (2014–2017), funded by the World Bank, provided a basic package of services free of charge in all PHC2s through a voucher system for every woman attending the health center for antenatal, delivery, or postnatal care (first six weeks), and children under the age of five with any illness (see [43] for more details). The project also included training, support for child vaccination in remote areas, and the provision of some equipment to health centers. The Mikolo project (2014–2018), funded by USAID, provided support to a network of 150 CHWs in the fokontany located more than 5 km from a health center in eight communes in Ifanadiana, four of which were in the HSS catchment and four in the comparison area. The project provided annual trainings and periodic supervision, along with equipment, supplies and an initial stock of medicines to each CHW. The ACCESS project (2019–2023) funded by USAID, continued the Mikolo community health efforts, and included support to primary care centers, with a focus on IMCI and maternal and reproductive health services, as well as additional support for HMIS and the procurement pillars of the health system. The main difference between the initial HSS catchment and the rest of the district was the duration of implementation of the HSS intervention at endline by the MoPH-Pivot partnership (9 years versus 2–6 years). Specific differences between catchments are described in Table 1.

### Population survey data: The IHOPE cohort

A longitudinal cohort study known as the Ifanadiana Health Outcomes and Prosperity longitudinal Evaluation (IHOPE cohort), was initiated in 2014 to collect demographic, health and socio-economic data from a representative sample of 1,600 households in Ifanadiana district over time [9]. Questionnaires were primarily adapted from the DHS [44]. The Madagascar National Institute of Statistics (INSTAT), which implements all major national health surveys in the country, was responsible for data collection, survey coordination, training, and oversight. A two-stage sampling process stratified the district by the initial HSS catchment and the rest of the district. Eighty clusters – half from each stratum – were selected at random from enumeration areas mapped during the 2009 census, and households were then mapped within each cluster. Twenty households were selected at random from each cluster.

The first wave of data collection was conducted between April and May of 2014. Individual face-to-face interviews were conducted with women aged 15–49 years and men aged 15–59 years (usual residents or visitors) in 1,522 of the sampled households (95.1% response rate). The original 1,600 households were revisited between August and September 2016 and again between April and June of 2018, 2021, and 2023; any missing or refused households were replaced with others from the same cluster using a predefined random replacement list. Response rates during follow-up survey waves varied between 94.5% and 95.9%. All residents, including children, had weight and height measured (or length, in the case of infants). Data collected in the questionnaires included, among other variables, household composition (size, gender, ages); indicators of socio-economic status (education, employment, household durable assets); recent illness (in the last 30 days) and care-seeking for all household members; preventive behavior (bed net ownership, access to water and sanitation); women’s reproductive history and care-seeking behavior for reproductive health; children’s health, development and care-seeking for illness; adult, maternal, and child mortality.

French and Malagasy questionnaires used in the cohort, along with data collection protocols, were standardized and validated for Madagascar through prior national surveys conducted by INSTAT. The study received ethical approval from the Madagascar National Ethics Committee and Harvard Medical School IRB. All adults (≥15 years) provided verbal consent for the in-person interview and anthropometric measurements. For children ≤ 5 years of age, consent was provided by a parent or guardian. Further details on data collection and survey design are available in [9]. Data were entered using CSPro.

### Health system utilization data

We obtained aggregated monthly utilization data for the period of January 2013 to December 2023 from the MoPH for 15 PHC2 and 6 PHC1s in Ifanadiana district. These data capture the number of new individuals attending each health center for outpatient consultations or maternal care. Because the HSS intervention began in early 2014 (shortly after the baseline survey) and affected utilization rates that year, we included health system utilization data from 2013 to establish a true pre-intervention baseline. These data were available from the health centers’ monthly reports to the district (RMA), which were compiled by MoPH staff from the health centers’ registers every month. Starting in May 2015, the MoPH revised its method for estimating and reporting outpatient utilization rates. Thus, all subsequent outpatient utilization data were gathered directly from the registers to ensure consistent estimates across the full 2013–2023 period.

To assess changes in geographic access to primary care, we analyzed both district-wide health center catchment data and data disaggregated to the smallest administrative unit—fokontany—for the initial HSS catchment area during the 2018–2020 period. Fokontany comprise one or several villages, and are located at varying distances from the nearest health center (0–20 km). To obtain the number of consultations at the PHC-level by fokontany, we first digitized individual-level data from the health center registries for all outpatient visits in the district. For each patient (new visits; follow-up excluded), information included the age, fokontany of residence and some diagnosis data. We also obtained consultation data from the monthly reports of community health sites supported by the MoPH-Pivot partnership for the 80 fokontany in the initial HSS catchment, available at the fokontany level. As a component of the HSS intervention, health system data quality was ensured with support from the NGO’s monitoring and evaluation team and through joint MoPH-Pivot field supervisions [36].

Population estimates for each health center catchment area per year (for aggregated data) or each fokontany (for disaggregated data) were derived from the two national censuses, conducted in 1993 and 2018 by INSTAT, applying a constant annual growth rate. Consistent with MoPH estimates, populations of children under five, expected number of pregnant women, and expected number of deliveries were set at 18%, 4.5%, and 4% of the total population, respectively. Finally, information on key dates of the implementation of health system strengthening components in each supported commune (such as the start of the user-fee exemptions, support to the community health program, or support to the maternal health program) were obtained from internal records within the NGO.

### Analysis of coverage and mortality rates from cohort data

Under-five, infant and neonatal mortality were estimated at the population level using the synthetic life-table method for DHS surveys [45]. These were defined as the probability of death before age 60 months, 12 months and 1 month per 1,000 children born alive, respectively. For each survey wave, we used information from children born in the five years prior to the survey. For each survey year, mortality rates were separately estimated for the entire district, and for the HSS catchments (initial HSS catchment versus rest of the district). From these estimates, absolute changes from the 2014 baseline were then calculated for each mortality indicator and population group.

Coverage indicators were estimated using standard definitions for DHS surveys [45]. Vaccination coverage was defined as the proportion of children aged 12–23 months who received all recommended vaccines: 3 doses of polio and diphtheria–tetanus–pertussis (DTP), one dose of Bacillus Calmette–Guérin (BCG) and measles. Care-seeking for illness was estimated as the proportion of children under five who had fever, acute respiratory infection, or diarrhea in the two weeks prior to the survey and sought medical care at a public health provider (hospital, health center or community health worker). For care seeking of individuals of all ages, a four week recall period for illness was used. To evaluate the impact of the HSS intervention on maternal health service coverage, indicators were estimated for the most recent pregnancy resulting in a live birth during the two years preceding the survey: prenatal care (first and fourth visit), delivery at a public health center and postnatal care. In addition, to track a summary indicator of maternal, neonatal and child (MNCH) coverage, we estimated a co-coverage index (five or more interventions) for women and children under five [46].

The impact of the HSS intervention on coverage indicators was modeled from individual-level data using multivariable logistic regressions (binomial model with logit link), controlling for relevant factors and accounting for survey weights, using the following formula:


$$\mathrm{\backslash footnotesize}Y\;=\;\alpha \;+\;\beta_{\text{1}}\;year\;+\;\beta_{\text{2}}\;catchment\;+\;\beta_{\text{3}}\;facility\_HSS\;+\;\beta_{\text{4}}\;program\_HSS\;+\;\beta_{\text{5}}\;voucher\;+\;\beta_{\text{6}}\;covid-\text{1}\text{9},$$ (1)

where *Y* represents the predicted population-level coverage; *year* is the number of years since the baseline survey*; catchment* reflects whether the household of the individual was part of the initial HSS catchment, to account for baseline differences in these two areas; *facility_HSS* reflects whether the household was in a commune where the HSS intervention was implemented at the facility level (including, but not limited to, reimbursement of user fees and readiness support), which changed over time as the HSS catchment expanded; *program_HSS* reflects whether the household was in a commune receiving support to a specific program affecting a subset of indicators, namely the community health program, with potential impact on care seeking indicators, or the maternal health program, with potential impact on maternal care indicators – these also changed over time as the HSS catchment expanded but all were implemented after initiation of the facility-level HSS; *voucher* reflects whether the household was in a commune where the voucher program funded by the World Bank for maternal and child care was implemented (see “Study site and HSS intervention”); *covid-19* reflects whether the covid-19 pandemic was occurring at the time of the survey (2021 follow-up) to account for a potential change in care seeking during this period. All these variables (except for *year*) were included as binary variables (0 when program or COVID-19 period not present in a particular commune and year, or 1 otherwise). The coefficient *β*_1_ is the yearly change in the study area independent of the intervention, *β*_2_ is the baseline difference between the initial HSS catchment and the rest of the district, *β*_3_, *β*_4_, *β*_5_, and *β*_6_ are the level of change associated with HSS support at the facility level, with HSS support to specific programs (community health or maternal health depending on the indicator), with the World Bank voucher program, and with the COVID-19 period, respectively. This design is equivalent to an interrupted time series analysis with control groups. Results are reported as adjusted odds ratios (OR).

All analyses were performed using R statistical software, version 4.2.1 [47]. All population-level analyses were done using survey commands available in the R-package *survey* and appropriate sampling weights [48], with the exception of population-level mortality rates and associated 95% confidence intervals, which were calculated with SAS 9.3 [49]. Sampling weights were calculated for household, women’s and men’s surveys, to adjust for unequal probability of selection due to stratification and non-response.

### Analyses of healthcare utilization from health center and community health data

To study the impact of the HSS intervention on consultation rates, aggregated health center data for the 2013–2023 period were used to model changes in monthly per capita utilization rates for maternal health services (antenatal care, first and fourth visits; deliveries, and postnatal care) and for outpatient care for any illness (all patients and children under five). For this, a negative binomial mixed-effects regression model – analogous to the analyses of survey data described above – were conducted for each indicator, with a random intercept for each health center. We applied interrupted time-series analysis with control groups, incorporating explanatory variables for facility-level and program-specific HSS support (i.e., maternal care and community health programs, depending on the indicator). The model also controlled for secular trends in the study area, baseline differences between the initial HSS catchment and the rest of the district, for the effect of the World Bank voucher program (2014–2017), and the COVID-19 pandemic (2020–2021). Health center catchment population was log-transformed and included as an offset. A 1-month lag in consultation data for each health center was included to control for temporal autocorrelation. Supplementary analyses were conducted to examine the slope of changes (progressive change over time) associated with facility-level HSS support and the World Bank voucher program, after adjusting for associated level changes (S2 and S3 Tables). Results are reported as adjusted relative changes. Statistical models were performed using the R package *lme4* [50].

To understand geographic trends in primary care utilization at different levels of care, individual-level data from health center registers were aggregated into consultations per capita-year for each of the 80 fokontany in the initial HSS catchment during 2018–2020, and compared with community health data from the same fokontany and period. The *Open Source Routing Machine *(OSRM)** engine was used to accurately estimate the shortest path between all buildings in each fokontany and the nearest health center. For this, the entire district was previously mapped on *OpenStreetMap*, resulting in over 23,000 km of footpaths, more than 100,000 buildings and 5,000 residential areas mapped (see [42] for details).

### Analysis of geographic and economic inequalities in healthcare coverage from cohort data

First, changes in the geographic distribution of coverage over time in Ifanadiana were assessed for selected indicators. Average values for the 80 geographic clusters in the IHOPE cohort were estimated, each of which included 20 households and approximately 100 individuals. Using the spatial location of each cluster, a raster surface of the entire district was generated to improve visualization of results. This was achieved through inverse distance weighted interpolation of the empirical Bayes estimates of the average coverage value of each cluster, using the R packages *spdep* and *gstat*.

Second, trends in economic and geographic inequalities within the HSS catchment were assessed for each coverage indicator over time. To estimate economic inequalities, a household wealth index was constructed using principal components analysis of household assets following standard DHS methods [8]. To estimate geographic inequalities, OSRM was used to estimate the shortest path between the villages in each cluster and the nearest health center, as explained above. For each indicator, we estimated wealth- and geographic-specific composite indicators of inequality, namely the relative concentration index (RCI) and slope index of inequality (SII) [51,52]. Lower values of the RCI or SII over time indicate reductions in relative or absolute inequalities, respectively.

Third, trends in self-reported barriers to seeking care were estimated in the initial HSS catchment and the rest of the district. Individuals of all ages in the IHOPE cohort surveys who reported being ill but did not seek care at a health facility were asked to provide their primary and secondary reasons for not seeking care (this information was not available for 2014). Reasons were classified into the following categories: no barrier (“not severe enough” or no reason reported); knowledge barrier (“did not think they could help me”, “did not know that a treatment existed”); health system barrier (“lack of confidence in health staff”, “health staff often absent”, “health staff is rude”, “health personnel asks for additional money”); financial barrier (“impossible to stop work”, “too expensive”); geographic barrier (“too far away or hard to reach”); or COVID-19 (“facility closed due to COVID-19”, only in 2021). Percentages of each barrier were estimated out of all primary and secondary reasons.

## Results

### Changes in child mortality rates at the population level

The results show clear signals of reductions in neonatal, infant, and U5 mortality rates following implementation of the HSS intervention. The number of children under-five used in mortality analyses was constant across survey waves: 4,063 children for 2014, 4,037 children for 2016, 3,788 children for 2018, 4,380 for 2021 and 3,983 for 2023. Across the district as a whole, the average U5 mortality rate did not consistently decrease during 2014–2023, while infant and neonatal mortality increased, patterns which are consistent with national trends observed in Madagascar from surveys over the same period (Fig 1). However, when trends were split by the initial HSS catchment and the rest of the district, a striking difference emerged whereby measures of child mortality in the initial HSS catchment consistently decreased for all child mortality indicators by 20–30 deaths (per 1,000 live births) as compared to the 2014 baseline, with opposite trends for the rest of the district (Fig 1). While rates at baseline in the initial HSS catchment were roughly twice the national average, all child mortality rates had converged with national averages by the end of the study period (Fig 1).

The initial HSS catchment had lower mortality rates at baseline than the rest of the district. The initial HSS catchment was better-off in socio-economic characteristics at baseline (e.g., access to electricity, and sanitation), and worse-off in others (e.g., more single parent households and a higher proportion of the population in the lowest wealth quintile). However, these characteristics remained largely stable during the study period (S2 Table) and were not accompanied by significant differences in healthcare access at baseline between the two areas (S3 Table). The comparison area (“rest of the district”) consists of 11 communes that received HSS support primarily starting in 2021. Following this expansion, under-five and infant mortality declined in the rest of the district after 2021, but neonatal mortality increased.

### Impact of HSS on health center utilization and population-level coverage of MNCH indicators

Statistical models of both health center utilization and population-level coverage yielded consistent results, revealing significant positive effects on indicators of child, adult, and maternal care after accounting for multiple confounders, including the expanding HSS catchment.

Time series analyses of monthly health center utilization (S1 Fig) revealed large and statistically significant effects of facility-level HSS (*e.g.,* improvements in readiness, clinical programs, removal of user fees) on outpatient consultations (Table 2), both for children under five (rate ratio 1.48, 95% CI [1.37, 1.61]; *p* < 0.05) and individuals of all ages (rate ratio 2.14, 95% CI [1.98, 2.32]; *p* < 0.05). Specific support to the community health program (“Program-specific HSS support”) was associated with a small but significant reduction in health center utilization. For maternal care consultations, both facility-level HSS and program-specific support to the maternal care program were associated with statistically significant increases in utilization (Table 2), although the cumulative impact associated with both of these interventions (approximately 25% increase) was smaller than in analyses of population-level coverage (Table 3). No statistically different baseline differences in utilization were observed between the intervention and comparison areas.

**Table 2:** Impact of health systems strengthening on per capita health center utilization rates for child, adult and maternal care from health system patient data (multivariable negative binomial mixed-effects model,^1^ one per indicator). Results are expressed as rate ratio (95% Confidence intervals).

Indicator	Baseline differences between catchments	Changes per year in Ifanadiana district	Facility-level HSS	Program-specific HSS support^2^	World Bank voucher program	Covid-19 period
**Child and adult care**						
Outpatient visits – All ages	0.98 (0.64, 1.5)	0.7 (0.64, 0.78)***	2.14 (1.98, 2.32)***	0.77 (0.7, 0.84)***	1.06 (1, 1.13)	1.02 (0.95, 1.09)
Outpatient visits – Children under five	0.8 (0.57, 1.13)	0.72 (0.65, 0.8)***	1.48 (1.37, 1.61)***	0.86 (0.78, 0.95)**	1.22 (1.14, 1.3)***	1.08 (1, 1.16)*
**Maternal care**						
Prenatal care (first visit)	0.91 (0.66, 1.26)	1.07 (0.98, 1.17)	1.12 (1.05, 1.2)***	1.12 (1.05, 1.2)**	1.15 (1.09, 1.22)***	1.04 (0.98, 1.11)
Prenatal care (four visits)	1.06 (0.66, 1.7)	0.67 (0.58, 0.77)***	1.13 (1.02, 1.24)*	1.18 (1.06, 1.3)**	1.26 (1.16, 1.36)***	0.84 (0.76, 0.92)***
Deliveries	0.74 (0.52, 1.05)	0.65 (0.58, 0.72)***	1.17 (1.09, 1.26)***	1.06 (0.99, 1.14)	1.28 (1.2, 1.36)***	0.95 (0.89, 1.02)
Postnatal care	0.88 (0.57, 1.38)	0.14 (0.11, 0.17)***	1.33 (1.16, 1.53)***	0.89 (0.77, 1.02)	1.79 (1.6, 2)***	1.01 (0.89, 1.14)

^1^Model includes a random effect for each health center, a population offset, and controls for lagged consultations at each health center (month-1). Results of a model that includes both level of change and slope of change for the two programs are available in S4 Table.

^2^HSS support to community health program for child and adult care indicators; HSS support to maternal health program for maternal care indicators.

* *p*-value < 0.05.

** *p*-value < 0.01.

*** *p*-value < 0.001.

Statistical test: Wald *z*-test.

Abbreviations: HSS Health Systems Strengthening.

**Table 3:** Impact of health systems strengthening on population-level coverage indicators of child, adult and maternal care from household survey data (multivariable binomial regression model accounting for survey weights,^1^ one model for each indicator). Results are expressed as Odds Ratio (95% Confidence intervals).

Indicator	Baseline differences between catchments	Changes per year in Ifanadiana district	Facility-level HSS	Program-specific HSS support^2^	World Bank Voucher Program	Covid-19 period
**Child and adult care**						
All recommended vaccines (12–23 months)	0.71 (0.36, 1.43)	1 (0.92, 1.08)	1.05 (0.58, 1.9)	1.96 (1.14, 3.36)*	1.08 (0.76, 1.54)	0.71 (0.46, 1.11)
Child care seeking for illness (<5 years, public provider)	0.67 (0.41, 1.1)	1.02 (0.96, 1.08)	1.89 (1.19, 3)**	1.13 (0.66, 1.95)	0.93 (0.75, 1.15)	0.95 (0.56, 1.63)
Individual care seeking for illness last 4 weeks (public provider)	0.89 (0.57, 1.41)	1.13 (1.01, 1.26)*	1.84 (1.3, 2.59)***	1.31 (0.9, 1.91)	1.4 (0.7, 2.78)	1.45 (1.06, 1.98)*
**Maternal care**						
Antenatal care (1+ visit with skilled provider)	1.18 (0.59, 2.34)	1 (0.93, 1.07)	1.39 (0.93, 2.07)	2.61 (1.46, 4.68)**	1.75 (1.39, 2.22)***	1.03 (0.75, 1.42)
Antenatal care (4+ visits with skilled provider)	0.79 (0.45, 1.38)	1.1 (1.02, 1.18)*	1.23 (0.75, 1.99)	1.3 (0.79, 2.13)	1.26 (0.93, 1.73)	0.82 (0.6, 1.12)
Birth delivered at public health center	0.72 (0.34, 1.52)	1.02 (0.94, 1.11)	1.75 (0.98, 3.12)	2.14 (1.17, 3.92)*	1.38 (0.97, 1.97)	0.82 (0.56, 1.2)
Postnatal care (within 48 h with skilled provider)	0.72 (0.34, 1.55)	0.99 (0.9, 1.09)	1.74 (0.99, 3.06)	2.08 (1.14, 3.79)*	1.24 (0.86, 1.79)	0.84 (0.57, 1.23)
Co-coverage index (5+ interventions)	0.71 (0.34, 1.49)	0.99 (0.91, 1.08)	1.35 (0.82, 2.23)	2.23 (1.11, 4.5)*	1.24 (0.86, 1.8)	0.93 (0.62, 1.39)

1 Results of a model that includes both level of change and slope of change for the two programs are available in S5 Table.

2 HSS support to community health program for child and adult care indicators; HSS support to maternal health program for maternal care indicators.

* *p*-value < 0.05.

** *p*-value < 0.01.

*** *p*-value < 0.001.

Statistical test: Wald *t* test.

Abbreviations: HSS Health Systems Strengthening.

Analyses of population-level service coverage from the IHOPE cohort (S2 Fig) showed that facility-level HSS had the largest effects on care-seeking for individuals of all ages and for children under five (Table 3), being associated with a near doubling of the odds of care seeking at a public provider (Odds Ratio [OR] 1.84, 95% CI [1.3, 2.59] and 1.89, 95% CI [1.19, 3], respectively; *p* < 0.05). For maternal care, facility-level HSS had positive but not statistically significant effects (e.g., OR for birth delivered at public health center of 1.75, 95% CI [0.98, 3.12]). Instead, program-specific HSS support to the maternal care program was associated with more than double the odds of having at least 1 prenatal care visit with a skilled provider, a birth delivered at a health facility and a postnatal care visit within 48 h – as well as a doubling of the co-coverage index, a summary index of maternal, child and neonatal care coverage. Program-specific HSS support to the community health program was associated with a significant increase in vaccination coverage for children 12–23 months (OR 1.96, 95% CI [1.14, 3.36]; *p* < 0.05) and an increase, although not statistically significant, in individual or child care seeking with any public provider (hospital, health center, or CHW) (Table 3).

### Evolution of inequalities in population-level coverage indicators

**Fig 2:**
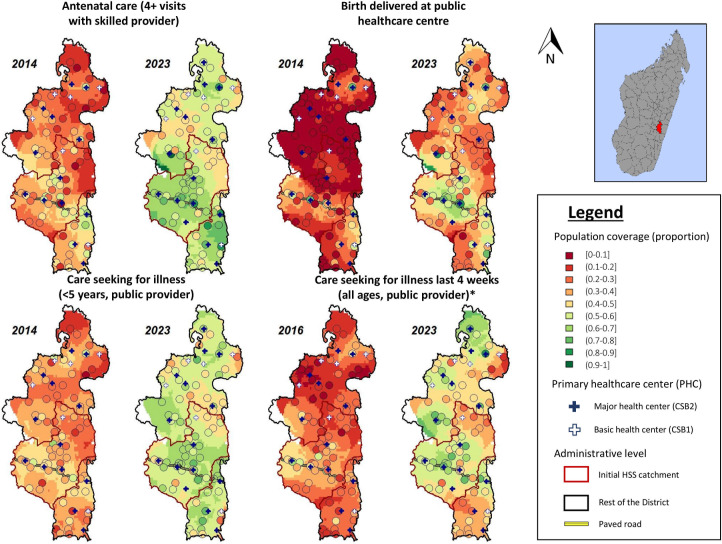
Spatial changes in selected population-level coverage indicators in Ifanadiana district, 2014–2023. Each map shows the weighted average for each of the 80 survey clusters (circles) in Ifanadiana district. Inverse distance weighted interpolation was used between clusters to improve visualization. Maps were created with R, with boundary data from OCHA (https://data.humdata.org/dataset/cod-ab-mdg) under a CC BY 4.0 License. *Data collection for care-seeking behavior for individuals of all ages began in 2016. Abbreviations: OCHA United Nations Office for the Coordination of Humanitarian Affairs (OCHA).

While the results above indicate increases in average health service coverage, spatial patterns over time for coverage indicators provided further insights into the impact of HSS in Ifanadiana district, and the gaps that remained in 2023 (Fig 2). Improvements were more spatially homogeneous for prenatal care (4+ visits) and for care-seeking of children under 5 than for the other indicators assessed, highlighting the role of CHWs in supporting referral and care delivery, respectively. Significant geographic gaps persisted for births delivered at health facilities and for care seeking of individuals of all ages, which can only occur at health centers or a hospital. In particular, coverage remained low in remote areas along the southern and eastern limits of the district, and in the catchment areas of basic health centers (PHC1), which had not yet been supported by HSS efforts (Fig 2).

**Fig 3:**
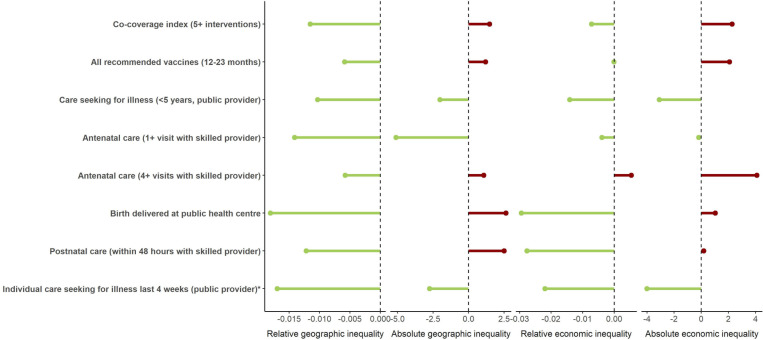
Relative geographic and economic inequalities were reduced for nearly all coverage indicators in the initial HSS catchment, 2014–2023, while changes in absolute inequalities were mixed. Figure shows the average annual change in relative inequalities (estimated as the relative concentration index) and absolute inequalities (estimated as the slope index of inequality) with respect to geography and wealth. For geographic inequalities, individuals were ranked according to their distance to the nearest health center. For wealth inequalities, individuals were ranked according to their household wealth score. The positive relative results compared to negative absolute results means that wealthier households and those that lived closer to health facilities experienced larger absolute increases in access to care, but poorer households that lived further from health facilities had greater proportional increases. *Data collection for care-seeking behavior for all ages began in 2016.

In the initial HSS catchment, both economic and geographic inequalities in healthcare coverage (both relative and absolute) were reduced for all ill patients seeking care, as well as U5 specifically (Fig 3). For nearly all other coverage indicators, relative inequalities declined in the initial HSS catchment, while absolute inequalities had mixed results (Fig 3). These results were consistent with the reasons for not seeking care at a health facility reported by individuals of all ages, where geographic and financial barriers were cited progressively less often over time in the initial HSS catchment, but changed little in the rest of the district (S3 Fig).

### The role of community health in reducing geographic barriers to care for children under five

**Fig 4:**
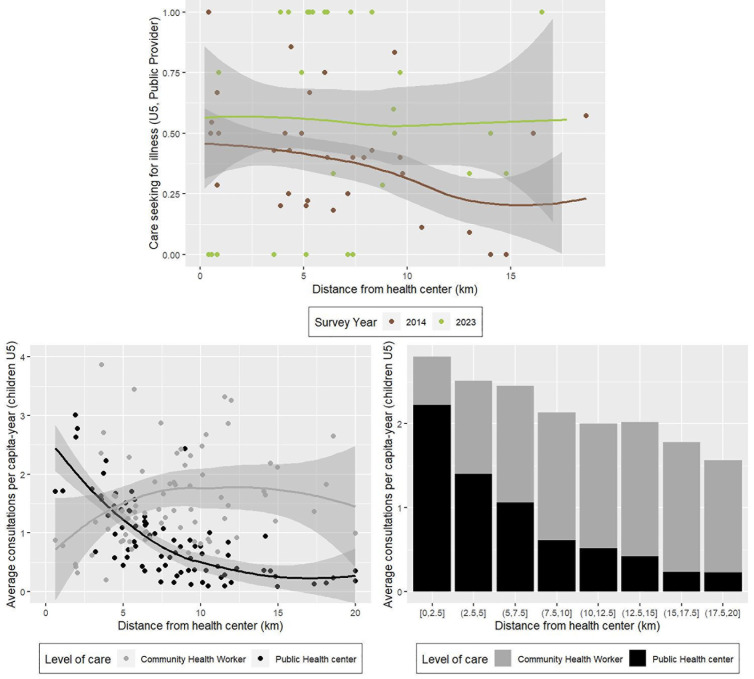
Household surveys (top row) and health system patient data (bottom row) indicate that reductions of geographic barriers through community health contributed to substantial increases in service utilization for children under 5 in remote areas. Top panel shows changes in care seeking for child illness at a public provider (health facility or CHW) from 2014 to 2023. Each dot represents the weighted average for each of the 80 clusters in the household survey, and the solid line represents a LOESS non-linear smooth of the trends and 95% confidence intervals. Bottom panels represent the split between health center and CHW utilization, estimated from health system data for the 80 fokontany in the initial HSS catchment during 2018–2020. Each dot represents the average annual per capita consultation rate for each fokontany (left), which is aggregated by distance categories (right). Abbreviations: CHW community health worker; HSS health systems strengthening; LOESS locally estimated scatterplot smoothing; U5 under five.

Geographic analyses of care-seeking behavior and health system utilization for children under five in the initial HSS catchment revealed an improvement in geographic access to care following the strengthening of the community health program (Fig 4). Survey results showed that, while care-seeking at a public provider (which includes both health facilities and community workers) initially (in 2014) declined with increasing household distance from health facilities, this effect disappeared by 2023 following HSS support, driven by larger improvements in care-seeking among more remote populations (Fig 4). Characterizing primary care consultations by level of care provided further insights into the relative contribution of CHWs and health facilities towards overall healthcare access for different geographic groups. Fig 4 shows that health center utilization declined exponentially as distance from health facilities increased. This distance decay was largely offset by increased CHW utilization along the same distance gradient. As a result, overall health system utilization for children under five remained above 2 consultations per capita-year across nearly all distances from health facilities (except for those further than 15 km, where it ranged from 1.5 to 2 consultations per capita-year). Health center consultations represented nearly 80% of primary care consultations for children under five living within 2.5 km of a health center, but less than 15% of primary care consultations for children living further than 15 km from a health center (Fig 4).

## Discussion

Why do so many children die each year in the face of abundant research suggesting that such deaths are preventable with known solutions? The challenge is that even simple solutions depend on broader, complex health systems that are difficult to change and to rigorously evaluate under real world conditions [53]. In addition to human resources, logistics, and infrastructure, there are political and social considerations across heterogeneous communities, partners and institutions. Rather than being discrete interventions with well-defined beginning and end points, lasting health system improvements must grow and adapt over time. Unambiguous empirical evidence on how mortality reductions can be achieved via specific health system interventions has accordingly been elusive.

Using a district-representative longitudinal cohort, we show the impact on population health of the first decade of implementation of a health system strengthening intervention in a rural district of Madagascar that had some of the worst indicators in the country, where U5 mortality at baseline was over twice the national average [54]. Consistent with larger trends observed for the country, child mortality rates worsened in the comparison group during the study period. In the intervention area, in contrast, these rates declined relatively rapidly, eventually converging with national trends. The intervention was associated with statistically significant increases in service coverage and primary care utilization during the same period, as well as reductions in geographic and financial barriers to care. By measuring both indirect and direct impacts of HSS on population health, specifically childhood mortality rates, this study shows how interventions strengthening health systems in low resource settings improve health system performance in ways that can explain corresponding decreases in population-level mortality rates.

Although there are standardized ways of measuring child mortality, there remains no gold standard for measuring population-level mortality impacts of HSS interventions that is comparable to randomized controlled trials (RCTs) of specific interventions on enrolled study participants [53]. An integrated health system intervention that is localized geographically and implemented under quasi-experimental conditions offers several distinct advantages. It holds promise for identifying mechanisms based on detailed local understanding of programs that can be difficult for large-scale retrospective policy analysis with low-resolution data in the presence of unknown confounders. However, there are fundamental tensions between programmatic priorities to maximize reductions in child mortality and study design considerations to maximize clarity of the statistical signal. Narrowly prescribed interventions that lend themselves to controlled experiments (such as RCTs) have less ability to impact population level mortality than a larger suite of medical interventions [53]. However, rolling out more services depends on more complex systems of support, more factors that are unknowable at the outset, and local adaptation over time [53].

In optimal control theory, this challenge is known generally as the dual control problem, which states that for dynamic systems where underlying parameters are not precisely known in advance, there is a trade-off between system identification (learning) and system control (management) [55]. The dual control problem is faced in many contexts, such as adaptive management in ecology, where the goal is similarly to improve the conditions for complex biological populations under natural conditions [56]. To address the issue, best practices in adaptive management recommend more comprehensive empirical approaches that combine *a priori* hypothesis-driven designs with broader passive surveillance, enabling a posteriori hypothesis testing as interventions learn and adapt [56–58]. We similarly approach this problem by using a broad array of data in a quasi-experimental design that includes adjacent intervention and comparison areas within a single district. The intervention expanded gradually over time, allowing for analysis of the effects as programs improved and the HSS catchment grew to eventually cover the entire district.

Other efforts that have focused on bottom-up improvements in community-based health systems have shown strong evidence of increases in healthcare access and coverage, with encouraging evidence of impacts on population health, but with critical data gaps that leave unresolved questions around mortality effects [25,28,59]. The Accelerated Child Survival and Development Programme in Ghana, Benin, and Mali, and an integrated community health program in Togo, reported decreases in mortality rates, but these trends did not differ from national trends [8,28]. A district level HSS intervention in eastern Rwanda that included community health saw under-five mortality decline (229.8 to 83.2 deaths per 1,000 live births in 5 years) at more than twice the rate of the rest of rural Rwanda [31]. However, limited baseline data and absence of prospective comparison groups, alongside major socioeconomic and demographic changes in Rwanda during that period, constrain causal interpretation. In peri-urban Mali, a doorstep community health program saw under-five mortality rates drop to well below national levels (154–7 deaths per live birth) after seven years [32], with the first two years alone declining by 86% (154–21). However, because under-five mortality is estimated from births in the previous five years, these estimates still included three years of data from the pre-intervention period, implying that other demographic changes must have contributed [32]. Compelling evidence comes from the 10-year evaluation of the Millennium Villages Project, which found a reduction in under-five mortality of 23 deaths per 1,000 live births across all 12 sites, using retrospectively identified comparison groups [29]. These results provide optimism for progress in the health-related SDGs, but are attributed to an integrated set of poverty reduction programs that include health, education, and agriculture among others, obscuring what can be attributed to healthcare.

In contrast to most sub-Saharan African countries, healthcare coverage and mortality rates have shown little progress, or have worsened, in Madagascar over the last 15 years. The two latest Madagascar DHS, conducted in 2009 and 2021, revealed that the percentage of pregnant women who gave birth at a healthcare facility increased only from 35% to 39%. Meanwhile, the proportion of children aged 12–23 months who received all basic vaccinations declined from 62% to 49% [60,61]. Care seeking at public facilities for common illnesses of children under five has remained stable at about 30% for diarrhea and about 40% for fever and acute respiratory infections [60,61]. These indicators are often lower among rural populations. Moreover, Madagascar is one of the few countries in the world where both malaria incidence and mortality have increased by over 75% as compared with 2015 levels [62]. This worsening of health indicators is due in part to persistent challenges in health financing and development assistance: Madagascar has among the lowest per capita health expenditures in the world, declining from $20 in 2009 to $16 in 2022 [63]. In contrast, the average per capita health expenditures of the least developed economies (UN classification) have risen steadily to nearly $50 in 2022; the average across sub-Saharan Africa reached $85 in 2022 [63]. Similar trends in development assistance for health have resulted in an increasing and substantial gap in aid (e.g., external health expenditure per capita) between Madagascar and the rest of sub-Saharan Africa [63].

The results from Madagascar presented here are important because of the unique combination of data and the overall context: (1) complete 10-year time series with a true baseline; (2) a longitudinal cohort that mitigates effects of changes in population structure, collected by independent specialists (the National Institute of Statistics); (3) representative samples from intervention and comparison groups; (4) patient data from the health system; and (5) a context that did not experience broader changes to socioeconomic and health conditions. As a result, we conclude that lower mortality rates in the initial HSS intervention area of Ifanadiana district can be explained by reductions in financial and geographic barriers (removal of user fees and expanded CHW support), alongside stronger, more integrated health systems at all levels of care (e.g., infrastructure upgrades, more trained personnel). These changes led to a doubling in care-seeking for illness in children and individuals of all ages, and to more modest but significant increases in maternal care coverage (Table 3). In contrast, the comparison area experienced mostly worsening health outcomes until this was reversed in 2021, when the HSS intervention was expanded to the rest of the district (Figs 1, S1 and S2). We show that support to the community health program contributed to increased care seeking and primary care utilization for children living further than 5 km from a health facility (Fig 4), but we did not find a significant impact of the program on care seeking overall in statistical models (Table 2). Instead, our results suggest that integration of HSS activities at all levels of care within a district health system had the strongest impacts on service coverage (Table 3).

**Fig 5:**
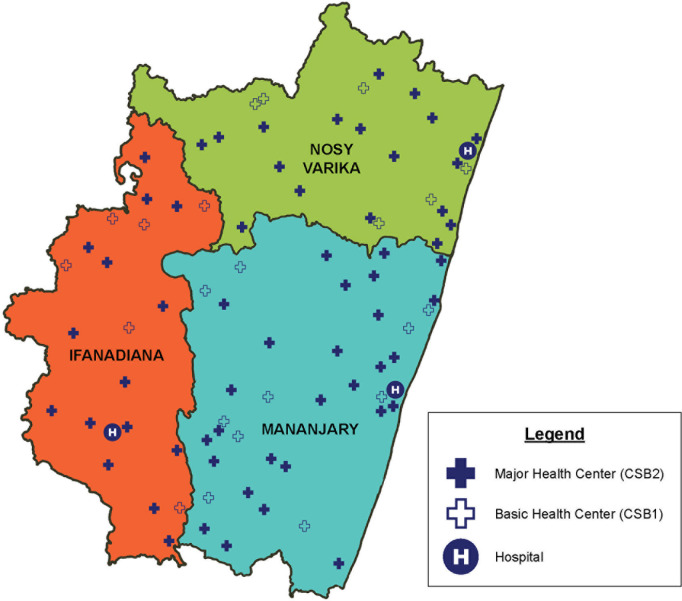
Geographic distribution of the regional HSS expansion in Vatovavy, Madagascar. The Region of Vatovavy comprises a population of approximately 1 million people in three districts. The expansion will be implemented in two main phases: in Nosy Varika district (green) from 2024 onwards, and in Mananjary district (blue) from 2027 onwards. Ifanadiana district (red) will continue receiving HSS support as described earlier. Core elements of the expanded model include: strengthening the community health program to reduce geographic barriers to care; social support and financial coverage for pregnant women and children to reduce financial barriers to care; and readiness support (human resources, infrastructure, etc.) to health centers and hospitals to improve service provision and quality of care. Boundary data were obtained from the United Nations Office for the Coordination of Humanitarian Affairs (https://data.humdata.org/dataset/cod-ab-mdg) under a CC BY 4.0 License.

A critical question, besides whether an intervention is effective, is its costs, scalability and sustainability. While formal cost-effectiveness analysis was not part of this study, supplementary costing analyses from the midpoint of the intervention (in 2018) estimated the total district health system in Ifanadiana, accounting for all payers, to cost approximately $60 per capita. This was funded from district-level investments of the Madagascar MoPH, NGOs, and international donors (S4 Table). Investments in service delivery (e.g., equipment, infrastructure, and commodities such as medicine) accounted for nearly half of all costs, while one-fifth of the investment was directed toward the workforce (S6 Table). Following a request from the Madagascar MoPH, a streamlined version of this HSS initiative is being expanded to the rest of the Region of Vatovavy, comprising a population of roughly 1 million people in 3 districts, including Ifanadiana (Fig 5). Core elements of the expanded model include: strengthening the community health program to reduce geographic barriers; social support and financial coverage for pregnant women and children to reduce financial barriers; and readiness support (human resources, infrastructure, etc.) to health centers and hospitals to improve service provision and quality of care [64]. Based on the protocol initially developed for Ifanadiana district [35], a population-representative baseline survey of 4,800 households (23,000 individuals) across the Region was conducted in 2023 and will be repeated routinely to measure the impact of this regional expansion on population health, offering a unique opportunity to further test the robustness of these findings at scale.

This study had several limitations. First, we used a quasi-experimental design where the intervention design was driven by programmatic decision-making. Neither the initial HSS catchment nor the phased geographic expansion of the intervention was randomized, raising valid questions about the counterfactual. While unobserved factors- such as shifts in the disease burden or the presence of other intervention – may have influenced outcomes, there were no statistically significant differences in baseline health system indicators, nor did socioeconomic indicators diverge over time. Moreover, although 2014 mortality rates in the initial catchment area were lower than in the rest of the district (RoD), they were higher than the national average (Fig 1A). Taken together, these local and national trends suggest that, in the absence of intervention, mortality rates would not have declined. In addition, contextual knowledge of the area allowed for important factors to be controlled for in the statistical analysis, such as other external programs and the COVID-19 epidemic. Second, integrated HSS interventions are inherently complex, involving multiple components that interact across levels of the health system (S1 Table), making it difficult to isolate individual effects. We used the presence of facility-level HSS implementation as a proxy for measuring the overall intervention exposure, while separately studying the effect of specific programs (community and maternal care programs) that were aimed at improving a subset of indicators. Future analyses could employ composite implementation strength scores [65] to more precisely assess how improving health system functionality relates to changes in coverage and mortality. Third, we assessed changes in child mortality from 5-year averages obtained from a synthetic cohort of the women’s birth history in each survey, in accordance with DHS methodology. While this allowed for comparison with national trends and with other studies, the overlapping nature of these time windows and populations prevented formal integration into the statistical modeling framework used for other indicators. Fourth, although we examined the main reasons why individuals did not seek care (S3 Fig), we lacked the data to deeply assess non-system factors such as cultural preferences for vaccination, home births, or use of traditional medicine. In addition, the statistical analyses on healthcare coverage and utilization were ecological in the sense that they relied on group-level rather than individual- or household-level confounders due to limited power, which may have introduced population-level bias. Finally, we did not have consultation data at the community level from before the HSS intervention started or outside of the HSS intervention area, which limited our ability to assess HSS impacts on geographic inequalities in utilization across all levels of care. However, population-level survey data was spatially explicit and covered the entire period, which showed improvements in both geographic inequalities and spatial distribution for many coverage indicators.

In conclusion, the many challenges to measuring impacts on population health in the real world have resulted in a persistent gap in the literature that calls into question the degree to which mortality rates can be meaningful targets for HSS interventions and decision-makers. Using a unique variety of data sources, we offer converging evidence that supports what many in the global health community have long advocated: that over time, strengthening local health systems with a strong foundation in community health, and removing financial and geographic barriers, can expand coverage of essential services and save lives. As countries like Madagascar strive to accomplish the SDGs, such integrated approaches, though difficult to implement and measure, are essential.

## Supporting information

S1 Fig Changes in health center utilisation rates in Ifanadiana district under HSS support, 2013–2023. Each dot represents the average annual per capita consultation rate for health centers in under the same catchment area. Vertical dashed lines represent the year when HSS support began in the initial catchment (green) and in the rest of the district (orange).
(DOCX)

S2 Fig Changes in population-level coverage indicators in Ifanadiana district under HSS support, 2014–2023. Each dot represents the weighted average for each survey year and catchment area. Vertical dashed lines represent the year when HSS support began in the initial catchment (green) and in the rest of the district (orange). More details on sample size and 95% confidence intervals are available in S3 Table.
(DOCX)

S3 Fig Primary and secondary reported reasons for not seeking care at a health facility, 2016–2023. It displays the percentage of responses out of all primary and secondary reasons that household members provided when they reported being ill in the previous 4 weeks but not seeking treatment at a health facility.
(DOCX)

S1 Table Summary of the HSS intervention carried out by the MoPH-Pivot partnership in Ifanadiana district in 2014–2023, based on guidelines from TIDieR (template for intervention description and replication). (DOCX)

S2 Table Impact of health systems strengthening on per capita health center utilization rates for child, adult and maternal care (multivariable negative binomial mixed-effects model, one per indicator). Results are expressed as Relative Change (95% Confidence intervals).
(DOCX)

S3 Table Changes in population-level coverage indicators in Ifanadiana district under HSS support, 2014–2023. (DOCX)

S4 Table Impact of health systems strengthening on per capita health center utilization rates for child, adult and maternal care (multivariable negative binomial mixed-effects model, one per indicator). Results are expressed as Relative Change (95% Confidence intervals).
(DOCX)

S5 Table Impact of health systems strengthening on population-level coverage indicators of child, adult and maternal care (multivariable binomial regression model accounting for survey weights, one per indicator). Results are expressed as Odds Ratio (95% Confidence intervals).
(DOCX)

S6 Table Costing of Ifanadiana district health system per capita. (DOCX)

S1 STROBE Checklist This checklist is licensed under the Creative Commons Attribution 4.0 International License (CC BY 4.0; https://creativecommons.org/licenses/by/4.0/). (DOCX)

## Abbreviations

HSS: health system strengthening
LiST: lives saved tool
RCI: relative concentration index
RCT: randomized controlled trial
SDG 3: sustainable development goal 3
SII: slope index of inequality
U5: under-five
UHC: universal health coverage
