# Supplementary material for: Changes in child mortality and population health following 10 years of health systems strengthening in rural Madagascar: A longitudinal cohort study
Source: PLoS Med. 2025 Oct 7;22(10):e1004549. doi: 10.1371/journal.pmed.1004549 (PMC12503271; doi:10.1371/journal.pmed.1004549)
Supplement: S1 Fig — Each dot represents the average annual per capita consultation rate for health centers in under the same catchment area. Vertical dashed lines represent the year when HSS support began in the initial catchment (green) and in the rest of the district (orange). (DOCX) [file pmed.1004549.s001.docx]

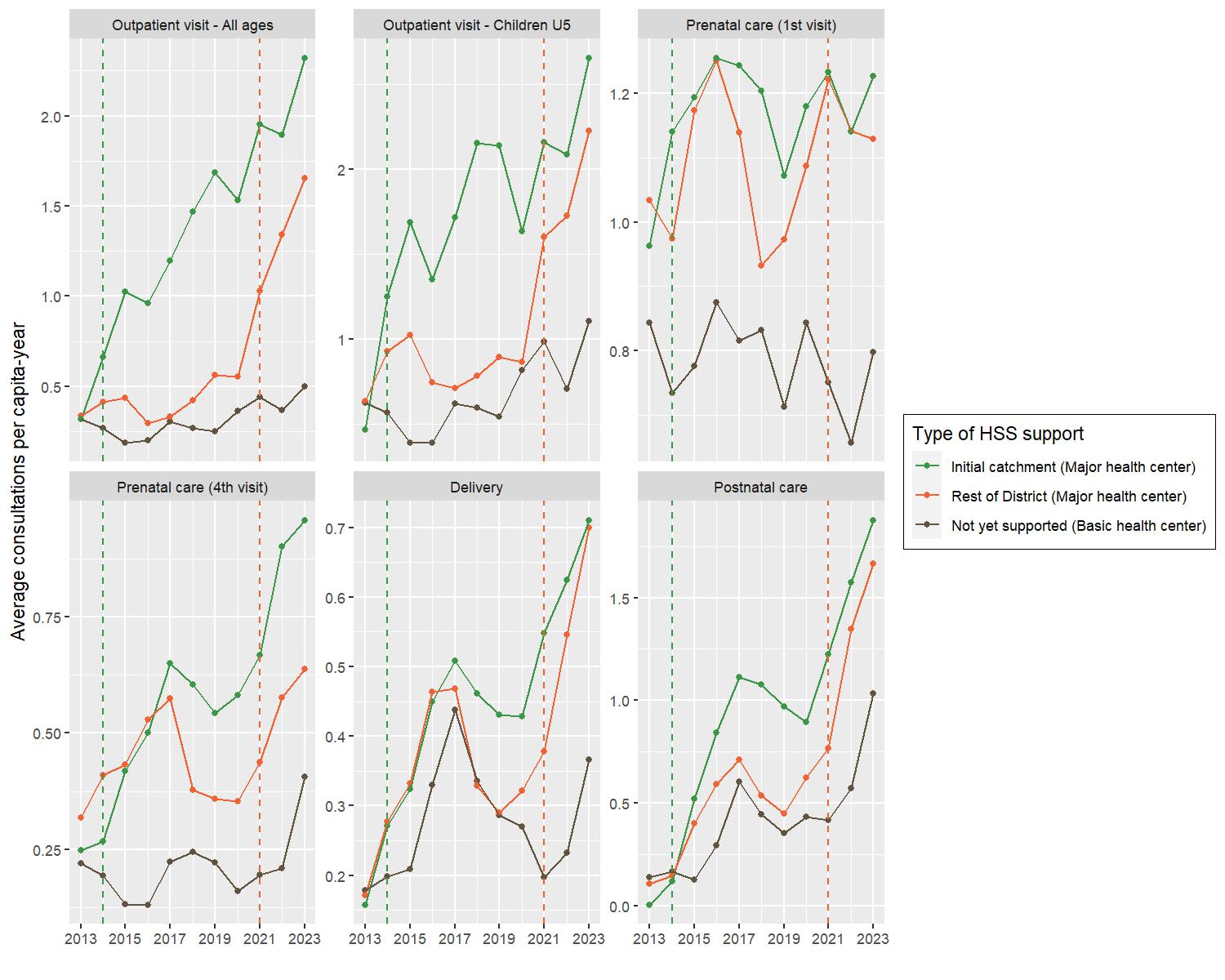


**Figure S1. Changes in health center utilisation rates in Ifanadiana District under HSS support, 2013-2023.** Each dot represents the average annual per capita consultation rate for health centers in under the same catchment area. Vertical dashed lines represent the year when HSS support began in the initial catchment (green) and in the rest of the district (orange)
