# Supplementary material for: Changes in child mortality and population health following 10 years of health systems strengthening in rural Madagascar: A longitudinal cohort study
Source: PLoS Med. 2025 Oct 7;22(10):e1004549. doi: 10.1371/journal.pmed.1004549 (PMC12503271; doi:10.1371/journal.pmed.1004549)
Supplement: S2 Fig — Each dot represents the weighted average for each survey year and catchment area. Vertical dashed lines represent the year when HSS support began in the initial catchment (green) and in the rest of the district (orange). More details on sample size and 95% confidence intervals are available in S3 Table. (DOCX) [file pmed.1004549.s002.docx]

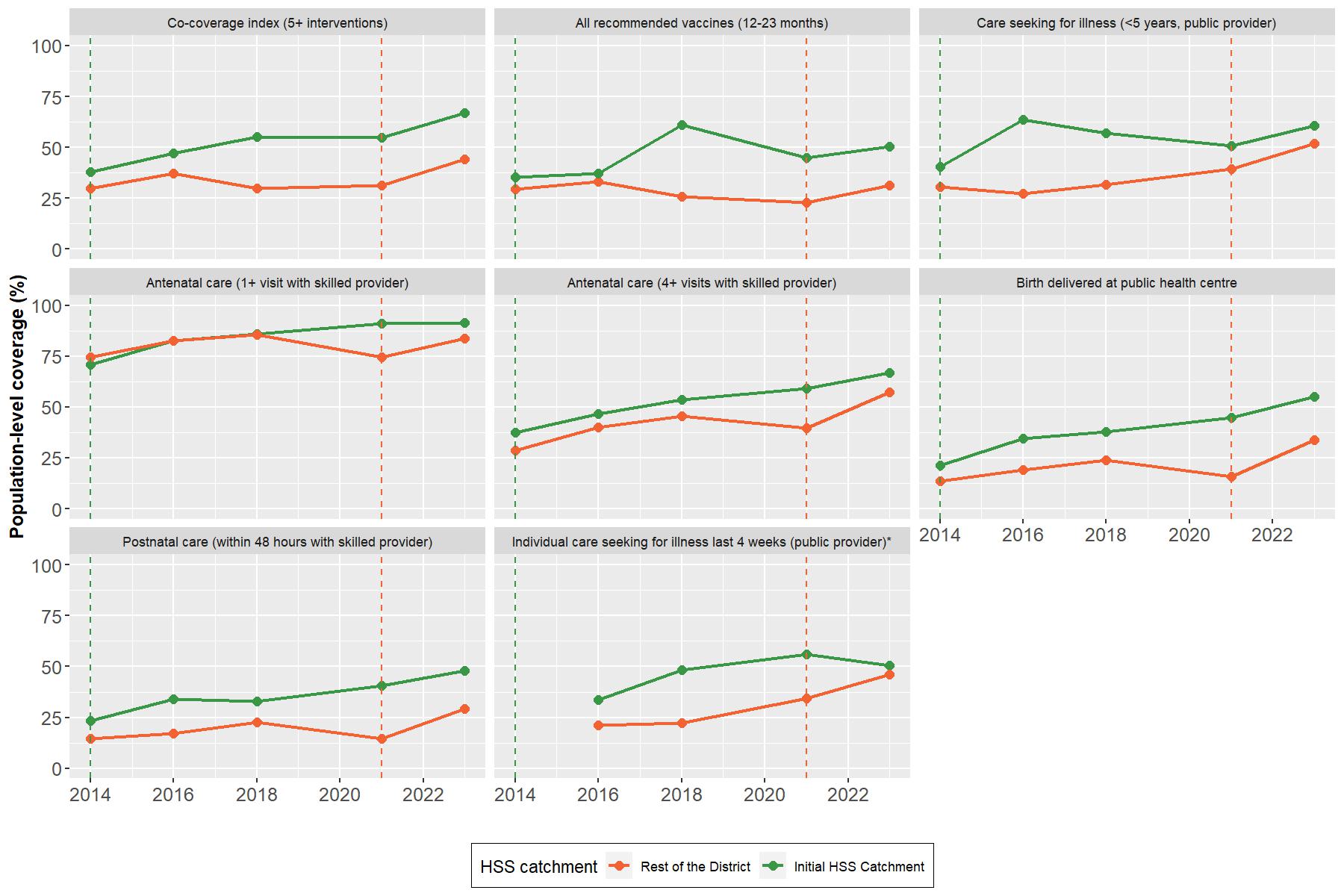


**Figure S2. Changes in population-level coverage indicators in Ifanadiana District under HSS support, 2014-2023.** Each dot represents the weighted average for each survey year and catchment area. Vertical dashed lines represent the year when HSS support began in the initial catchment (green) and in the rest of the district (orange). More details on sample size and 95% confidence intervals are available in Table S3.
