# Supplementary material for: Changes in child mortality and population health following 10 years of health systems strengthening in rural Madagascar: A longitudinal cohort study
Source: PLoS Med. 2025 Oct 7;22(10):e1004549. doi: 10.1371/journal.pmed.1004549 (PMC12503271; doi:10.1371/journal.pmed.1004549)
Supplement: S3 Fig — It displays the percentage of responses out of all primary and secondary reasons that household members provided when they reported being ill in the previous 4 weeks but not seeking treatment at a health facility. (DOCX) [file pmed.1004549.s003.docx]

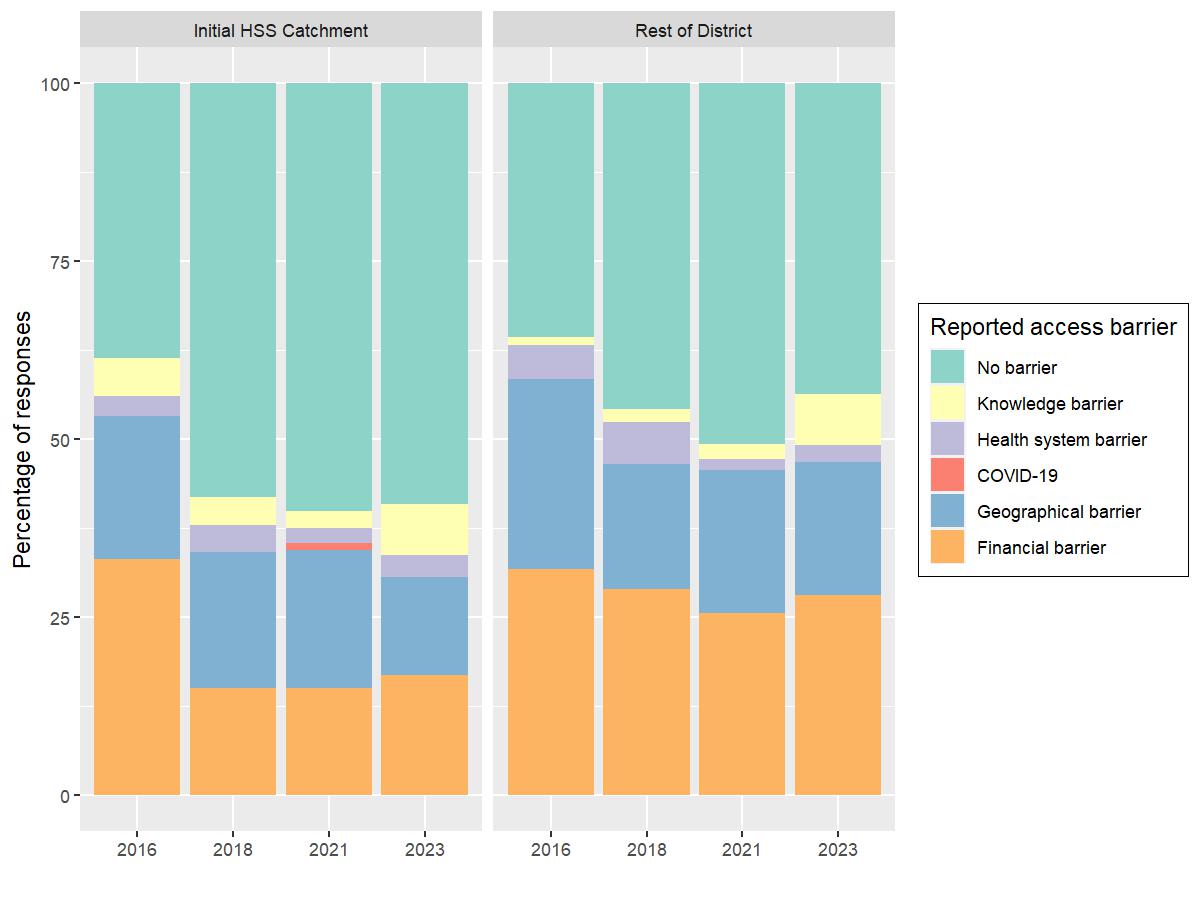


**Figure S3. Primary & secondary reported reasons for not seeking care at a health facility, 2016-2023.** It displays the percentage of responses out of all primary and secondary reasons that household members provided when they reported being ill in the previous 4 weeks but not seeking treatment at a health facility.
