# Supplementary material for: Changes in child mortality and population health following 10 years of health systems strengthening in rural Madagascar: A longitudinal cohort study
Source: PLoS Med. 2025 Oct 7;22(10):e1004549. doi: 10.1371/journal.pmed.1004549 (PMC12503271; doi:10.1371/journal.pmed.1004549)
Supplement: S1 Table — (DOCX) [file pmed.1004549.s004.docx]

**Table S1.** Summary of the HSS intervention carried out by the MoPH-Pivot partnership in Ifanadiana district in 2014-2023, based on guidelines from TIDieR (template for intervention description and replication).

| **1. BRIEF NAME** |
| --- |
| Integrated health system strengthening initiative in the district of Ifanadiana, Madagascar |
| **2. WHY** |
| ***GOAL/RATIONALE:*** Create a model public health district with universal access to care aimed at large-scale population health impact on mortality. Based on the World Health Organization’s building blocks of health system strengthening: 1) service delivery; 2) health personnel; 3) health information systems; 4) medicines and supplies; 5) financing; 6) leadership/governance. |
| **WHAT** |
| **3. *MATERIALS*** (by level of care and above enumerated building blocks)  **At district hospital level**  (1) *Service delivery:* Comprehensive modernization of infrastructure and equipment for the provision of services; increasing the hospital bed capacity from 55 to 61; modernization of the waste management system; specific renovations of the emergency and triage department, pediatric unit, hospitalization room, isolation room and laboratory; support for the provision of specific services, including emergency care and the provision of a network of 3 ambulances, with 24/7 coverage and Pivot paramedics for referrals; maternal and obstetric care; laboratory services have been enhanced to include a total of 54 tests, including microscopy and GeneXpert for tuberculosis; in 2023, the laboratory established microbiology services; social support has evolved to support all hospitalized and vulnerable patients; launch of an intensive care unit for severe acute malnutrition with complications.  (2) *Health personnel:* Staffing of health workers to achieve Ministry of Public Health standards through joint PIVOT-MoPH recruitment. Five waves of joint recruitment were carried out, including 9 doctors, 27 nurses and 5 midwives; 17 were integrated into the staff of the Ministry of Public Health for long-term employment; Pivot non-clinical staff comprising a team of 2 social workers, support staff (janitors, guards, etc.), a laboratory technician and an x-ray technician; ongoing mentorship and frequent training of medical staff in key clinical areas, such as emergency medicine and post-operative care.  (3) *Health Information Systems:* Creation of a hospital M&E team to monitor the progress of activities and improve the quality of HMIS data; implementation of a new approach to evaluating the operational capacity of health facilities in line with the recommendations of the Ministry of Public Health.  (4) *Medicines and supplies:* Supply chain management and reduction of stock outs, initially through frequent donations which evolved into a reimbursement program combined with pharmacy management training; provision of medical and non-medical equipment for service delivery, including full laboratory capacity. Cessation of Pivot functioning as CHRD pharmacy manager in 2024.  (5) *Financing:* Fully covered cost of outpatient and inpatient care for patients referred from district health centers and self-referred patients who required urgent hospital care (more than 76 000 patient visits between 2014 and the end of 2023); the cost of referral and healthcare at higher levels of care (e.g., a teaching hospital) is fully covered for services not available in the district hospital.  (6) *Leadership/governance:* Creation of a joint Pivot-MoPH executive committee for hospital management and transparency; creation of subcommittees for specific projects such as infection control or quality of care.  **At the health center level**  (1) *Service delivery:* Comprehensive renovations and/or expansion of infrastructure for service delivery in 19 health centers, including ensuring electricity, water, waste management/sterilization capacity and appropriate pharmaceutical conditions; provision of medical and non-medical equipment, including beds, wardrobes, furniture; support for the launch of specific services for the integrated management of childhood illnesses and malnutrition protocols for all children under 5 years old attending the health center; support labor and delivery services; ensure timely referrals and emergency care. Implementation of health programs linked to non-communicable diseases, notably diabetes and cervical and uterine cancer.  (2) *Health personnel:* Staffing through joint hiring by the MoPH and Pivot to align primary health care centers to Ministry of Public Health standards (1 doctor, 1 nurse, 1 midwife, 1 dispenser at the pharmacy, 1 support staff in each facility); 09 doctors, 43 nurses and 58 midwives were jointly recruited, 35 of whom are integrated into the MoPH; in target health facilities, recruitment exceeded standards and Pivot clinicians were present at all times (~2 clinicians per health center) to implement service delivery protocols (e.g. IMCI, malnutrition); training for medical staff (some district-wide) such as obstetric and neonatal care; ongoing supervision; mentoring in IMCI, malnutrition, prenatal and postnatal care, labor and delivery.  (3) *Health Information Systems:* Joint MoPH and Pivot training and supervision to improve HMIS data quality (district-wide); Transition to a new approach to evaluating the operational capacities of health facilities according to the MoPH directives; training of health workers on the electronic tool for reporting activities (RMAE).  (4) *Medicines and supplies:* Supply chain management and reduction of stock outs, initially through frequent donations which evolved into a reimbursement program combined with pharmacy management training.  (5) *Financing:* Essential medicines and consumables provided free of charge to all patients (over 1,000,000 patient visits between the October 2014 launch and the end of 2023).  (6) *Leadership/governance:* Close collaboration with district health officials for planning and implementation of activities.  **At the community level**  (1) *Service delivery:* Construction of 93 community health posts; provision of specific services for integrated management of childhood illnesses and malnutrition for every child under 5 years of age, community awareness, mass testing for select diseases, emergency care; mobile clinics with direct care delivery by Pivot clinicians every two months. In 2017, Pivot began supporting monthly supervision of CHWs in health facilities.  (2) *Health personnel:* 20 active community health supervisors – Pivot staff who train, oversee and conduct monthly supervision of approximately 195 community health workers at the health center and community-based supervision every two months; community training in IMCI provided to community health workers throughout the intervention area.  (3) *Health Information Systems:* Joint MoPH-Pivot training to improve the quality of HMIS community health data. Training of 138 community agents with the Commcare application; continuous support for community agents in the use of mobile technology.  (4) *Medicines and supplies:* Monthly supply and monitoring of stock of MNCH medicines and supplies, including commodities for diagnosis and treatment of malaria, oral rehydration salts, NSAIDs, antibiotics and zinc.  (5) *Financing:* The cost of MNCH drug stocks is fully covered; financial and non-financial incentives for CHWs and local leaders.  (6) *Leadership/governance::* Community engagement and participation (for example, community health posts are built by the community, with support from Pivot for roofing, painting, furniture and equipment).  **4. *PROCEDURES***  All interventions aimed to comply with existing protocols and standards of the Madagascar Ministry of Public Health. |
| **5. WHO PROVIDED** |
| **At district hospital level**  Clinicians from the MoPH provided the majority of service delivery. Pivot clinicians were integrated into the hospital staff and provide direct care like any other clinician during outpatient visits and clinical rounds, but also conduct frequent training. Pivot's non-clinical staff provided social support to vulnerable patients and helped manage the patient circuit to benefit from fee waivers (registration, validation).  **At the health center level**  Doctors and nurses from the MoPH provided the majority of services. According to the standards of the MoPH, each health center (CSB2) should have 1 doctor, 1 nurse, 1 midwife, 1 dispenser at the pharmacy, and 1 support staff.  **At the community level**  Two community health workers per fokontany (a group of villages, lowest administrative unit) provided basic MNCH care, supervised monthly by clinicians at their respective health center. Pivot mobile supervisor teams provided community-based mentoring and supervision of CHWs every two months. |
| **6. HOW (modes of delivery)** |
| Pivot employees worked in partnership with existing networks of MoPH clinicians and community health workers within existing public health settings. Where possible, as with supply chain management, leadership and financing, the intervention deliberately avoids the creation of parallel systems of care. |
| **7. WHERE** |
| **At district hospital level**  Pivot's initial catchment area included the only district hospital, located in the town of Ifanadiana. Most referrals to higher levels of care (tertiary) were sent to Fianarantsoa University Hospital (2 hours by car from the district hospital), and some to specialist facilities in Antananarivo (the national capital, about 1 day by car)  **At the health center level**  Comprehensive health center activities were implemented in all 21 level-two health centers in the district. Implementation began in the five communes closest to the hospital on the only asphalt road in the district (i.e. Ranomafana, Kelilalina, Ifanadiana, Tsaratanana, Antaretra) and expanded over time to the rest of the district. Throughout the project, all 21 health centers in the district received training, staff support to achieve Ministry of Health standards, and some access to the referral network (limited by accessibility).  **At the community level**  By the end of 2023, community activities had been deployed in the fokontany of eight communes of the district. In one commune, a more intensive proactive community health worker model was deployed. |
| **8. WHEN AND HOW MUCH** |
| All interventions were progressively rolled out over the study period.  The first intervention activities implemented (from April-May 2014, at the start of the study period) included the ambulance network, staffing of health centers and the district hospital, and the supply of medical equipment in four municipalities.  Renovation of health centers also began in April-May 2014 but the completion date varies according to each health facility.  The renovation of the emergency and triage unit and pediatric on-call at the district hospital was completed in early 2016.  The removal of user fees in health centers and hospitals began in October 2014; by July 2021, user fees had been removed at all level-two health centers across the district.  Implementation of IMCI and malnutrition protocols in all health centers began in October 2015.  Activities at the community level began in November 2015 in two municipalities, with an extension to eight communes in 2023.  First extension of the Pivot intervention zone at the level of health facilities in 2014, including total coverage of municipalities (15 CSB2) in 2023 |
| **9. TAILORING** |
| N/A |
| **10. MODIFICATIONS** |
| N/A; The intervention is progressively being implemented, as explained in section 8 (when and how much) |
| **HOW WELL** |
| N/A; The aim of this study was to investigate the evolution of access to strengthened primary care in Ifanadiana District. Full details of the impact analysis are available in the main text. |
