# Supplementary material for: Changes in child mortality and population health following 10 years of health systems strengthening in rural Madagascar: A longitudinal cohort study
Source: PLoS Med. 2025 Oct 7;22(10):e1004549. doi: 10.1371/journal.pmed.1004549 (PMC12503271; doi:10.1371/journal.pmed.1004549)
Supplement: S2 Table — Results are expressed as Relative Change (95% Confidence intervals). (DOCX) [file pmed.1004549.s005.docx]

**Table S2.** Socio-economic characteristics in the population of Ifanadiana District, 2014-2023

|  | 2014 | | | 2023 | | |
| --- | --- | --- | --- | --- | --- | --- |
| **Indicators** | **Ifanadiana district** | **Initial HSS catchment** | **Rest of District (RoD)** | **Ifanadiana district** | **Initial HSS catchment** | **Rest of District (RoD)** |
| Number of households surveyed | 1522 | 630 | 892 | 1534 | 637 | 897 |
| **Socio-economic characteristics** |  |  |  |  |  |  |
| Household size (N) | 5.4 | 5.3 | 5.5 | 5.0 | 5.0 | 5.0 |
| Female-headed household (%) | 17.8 | 20.8 | 15.7 | 23.1 | 27.2 | 20.4 |
| Primary occupation of household head is agriculture (%) | 84.7 | 72.1 | 92.6 | 79.8 | 70.9 | 85.6 |
| Access to improved sanitation (%) | 2.9 | 6.4 | 0.6 | 7.6 | 14.0 | 3.3 |
| Access to safe drinking water (%) | 14.9 | 30.7 | 4.4 | 20.7 | 33.1 | 12.4 |
| Households with at least 1 bed net (%) | 94.8 | 93.3 | 95.8 | 83.3 | 82.4 | 83.8 |
| Proportion of households in lowest wealth quintile | - | 10.4 | 23.4 | - | 11.4 | 24.6 |
