# Supplementary material for: Changes in child mortality and population health following 10 years of health systems strengthening in rural Madagascar: A longitudinal cohort study
Source: PLoS Med. 2025 Oct 7;22(10):e1004549. doi: 10.1371/journal.pmed.1004549 (PMC12503271; doi:10.1371/journal.pmed.1004549)
Supplement: S3 Table — (DOCX) [file pmed.1004549.s006.docx]

**Table S3.** Changes in population-level coverage indicators in Ifanadiana District under HSS support, 2014-2023.

| **Indicator** | **Catchment** | **2014** | | | **2016** | | | **2018** | | | **2021** | | | **2023** | | |
| --- | --- | --- | --- | --- | --- | --- | --- | --- | --- | --- | --- | --- | --- | --- | --- | --- |
| **Child and adult care** |  | **N** | **Mean (95% CI)** | **p-value** | **N** | **Mean (95% CI)** | **p-value** | **N** | **Mean (95% CI)** | **p-value** | **N** | **Mean (95% CI)** | **p-value** | **N** | **Mean (95% CI)** | **p-value** |
| All recommended vaccines (12-23 months) | Initial Catchment | 82 | 35,45 (21,68-49,22) |  | 84 | 37,17 (23,7-50,65) |  | 73 | 61,17 (47,77-74,58) |  | 58 | 44,85 (27,9-61,79) |  | 51 | 50,52 (31,49-69,54) |  |
|  | Rest of District | 152 | 29,42 (17,93-40,9) | 0,51 | 141 | 33,35 (22,56-44,15) | 0,664 | 120 | 26,01 (16,8-35,21) | <0,001 | 123 | 22,83 (13,63-32,03) | 0,022 | 97 | 31,4 (21,56-41,24) | 0,078 |
| Child care seeking for illness (<5 years, public provider) | Initial Catchment | 185 | 40,62 (30,4-50,84) |  | 103 | 63,62 (51,36-75,89) |  | 107 | 57,05 (44,78-69,32) |  | 66 | 50,78 (35,1-66,47) |  | 65 | 60,76 (46,28-75,23) |  |
|  | Rest of District | 443 | 30,53 (23,48-37,58) | 0,11 | 277 | 27,25 (19,29-35,22) | <0,001 | 234 | 31,6 (22,67-40,52) | 0,002 | 143 | 39,32 (25,33-53,31) | 0,291 | 127 | 51,88 (41,03-62,73) | 0,344 |
| Individual care seeking for illness last 4 weeks (public provider) | Initial Catchment | 0 | - |  | 915 | 33,71 (27,71-39,72) |  | 826 | 48,34 (38,13-58,55) |  | 863 | 56,07 (48,03-64,1) |  | 814 | 50,38 (43,79-56,96) |  |
|  | Rest of District | 0 | - | - | 1568 | 20,99 (15,72-26,25) | 0,003 | 1425 | 22,33 (16,92-27,74) | <0,001 | 1459 | 34,39 (28,23-40,55) | <0,001 | 1306 | 45,97 (37,39-54,56) | 0,428 |
| **Maternal care** |  |  |  |  |  |  |  |  |  |  |  |  |  |  |  |  |
| Antenatal care (1+ visit with skilled provider) | Initial Catchment | 182 | 70,83 (58,39-83,27) |  | 172 | 82,75 (72,37-93,12) |  | 160 | 86,05 (78,5-93,61) |  | 132 | 90,88 (85,04-96,72) |  | 105 | 91,49 (85,16-97,83) |  |
|  | Rest of District | 338 | 74,64 (67,3-81,98) | 0,6 | 335 | 82,63 (75,25-90,02) | 0,986 | 264 | 85,53 (79,8-91,26) | 0,914 | 261 | 74,41 (66,01-82,81) | 0,003 | 212 | 83,68 (77,08-90,28) | 0,122 |
| Antenatal care (4+ visits with skilled provider) | Initial Catchment | 182 | 37,27 (26,87-47,66) |  | 172 | 46,66 (33,05-60,26) |  | 160 | 53,46 (40,98-65,94) |  | 132 | 59,17 (46,19-72,16) |  | 105 | 66,68 (56,31-77,06) |  |
|  | Rest of District | 338 | 28,57 (20,72-36,41) | 0,19 | 335 | 40 (29,92-50,09) | 0,442 | 264 | 45,61 (35,6-55,61) | 0,34 | 261 | 39,55 (30,15-48,96) | 0,021 | 212 | 57,18 (45,97-68,38) | 0,227 |
| Birth delivered at public health center | Initial Catchment | 182 | 21,28 (12,8-29,76) |  | 172 | 34,67 (21,44-47,9) |  | 160 | 37,64 (27,26-48,03) |  | 132 | 44,73 (26,97-62,49) |  | 105 | 55,16 (41,64-68,67) |  |
|  | Rest of District | 338 | 13,66 (5,93-21,39) | 0,205 | 335 | 19,05 (11,38-26,71) | 0,039 | 264 | 23,98 (15,63-32,34) | 0,048 | 261 | 15,76 (8,38-23,14) | 0,002 | 212 | 33,71 (19,81-47,61) | 0,037 |
| Postnatal care (within 48 hours with skilled provider) | Initial Catchment | 182 | 23,45 (13,11-33,78) |  | 172 | 33,99 (21,03-46,95) |  | 160 | 32,91 (23,34-42,49) |  | 132 | 40,75 (23,6-57,91) |  | 105 | 47,87 (32,25-63,49) |  |
|  | Rest of District | 338 | 14,55 (5,43-23,67) | 0,217 | 335 | 16,98 (9,44-24,51) | 0,022 | 264 | 22,52 (15,19-29,85) | 0,092 | 261 | 14,52 (9,06-19,98) | 0,001 | 212 | 29,33 (17,1-41,56) | 0,071 |
| Co-coverage index (5+ interventions) | Initial Catchment | 78 | 37,85 (23,04-52,66) |  | 75 | 47,08 (31,74-62,42) |  | 73 | 55,06 (41,37-68,76) |  | 56 | 54,79 (38,09-71,48) |  | 48 | 66,88 (48,65-85,12) |  |
|  | Rest of District | 140 | 29,87 (17,18-42,55) | 0,424 | 140 | 37,36 (24,42-50,3) | 0,345 | 120 | 29,87 (19,21-40,54) | 0,007 | 117 | 31,17 (20,17-42,18) | 0,024 | 91 | 44,02 (28,75-59,28) | 0,073 |
