# Supplementary material for: Changes in child mortality and population health following 10 years of health systems strengthening in rural Madagascar: A longitudinal cohort study
Source: PLoS Med. 2025 Oct 7;22(10):e1004549. doi: 10.1371/journal.pmed.1004549 (PMC12503271; doi:10.1371/journal.pmed.1004549)
Supplement: S4 Table — Results are expressed as Relative Change (95% Confidence intervals). (DOCX) [file pmed.1004549.s007.docx]

**Table S4.** Impact of health systems strengthening on per capita health center utilization rates for child, adult and maternal care (multivariable negative binomial mixed-effects model^1^, one per indicator). Results are expressed as Relative Change (95% Confidence intervals)

| **Indicator** | **Baseline differences between catchments** | **Change over 10 years in study area** | **Facility-level HSS (level of change)** | **Facility-level HSS (Slope of change)** | **Program-specific HSS support^1^** | **World Bank voucher program (level of change)** | **World Bank voucher program (slope of change)** | **Covid-19 period** |
| --- | --- | --- | --- | --- | --- | --- | --- | --- |
| **Child and adult care** |  |  |  |  |  |  |  |  |
| Outpatient visits - All ages | 0.96 (0.63-1.46) | 0.66 (0.6-0.74)*** | 2.16 (1.92-2.43)*** | 1.01 (0.83-1.24) | 0.81 (0.74-0.89)*** | 0.42 (0.32-0.57)*** | 3.25 (2.25-4.68)*** | 1 (0.93-1.08) |
| Outpatient visits - Children under five | 0.79 (0.56-1.11) | 0.67 (0.6-0.75)*** | 1.44 (1.28-1.62)*** | 1.1 (0.9-1.34) | 0.9 (0.82-0.99)* | 0.51 (0.38-0.69)*** | 3.04 (2.08-4.44)*** | 1.06 (0.98-1.14) |
| **Maternal care** |  |  |  |  |  |  |  |  |
| Prenatal care (first visit) | 0.93 (0.68-1.29) | 1.1 (1.01-1.21)* | 1.19 (1.07-1.31)*** | 0.87 (0.72-1.06) | 1.09 (1.01-1.19)* | 1.46 (1.13-1.9)** | 0.75 (0.54-1.03). | 1.05 (0.99-1.12) |
| Prenatal care (four visits) | 1.09 (0.67-1.77) | 0.71 (0.61-0.82)*** | 1.22 (1.05-1.42)* | 0.82 (0.61-1.09) | 1.14 (1.01-1.29)* | 2.07 (1.41-3.03)*** | 0.54 (0.33-0.87)* | 0.85 (0.77-0.94)** |
| Deliveries | 0.73 (0.51-1.04) | 0.66 (0.58-0.74)*** | 1.14 (1.02-1.27)* | 1.07 (0.87-1.32) | 1.09 (1.01-1.19)* | 1.75 (1.32-2.31)*** | 0.66 (0.47-0.94)* | 0.95 (0.88-1.02) |
| Postnatal care | 0.7 (0.44-1.1) | 0.14 (0.11-0.17)*** | 0.86 (0.7-1.05) | 3.24 (2.14-4.9)*** | 1.21 (1.03-1.43)* | 9.79 (5.65-16.98)*** | 0.1 (0.05-0.19)*** | 0.95 (0.84-1.07) |

^1^ Model includes a random effect for each health, a population offset, and controls for lagged consultations at each health center (month-1)

^2^ HSS support to community health program for child and adult care indicators; HSS support to maternal health program for maternal care indicators
