# Supplementary material for: Changes in child mortality and population health following 10 years of health systems strengthening in rural Madagascar: A longitudinal cohort study
Source: PLoS Med. 2025 Oct 7;22(10):e1004549. doi: 10.1371/journal.pmed.1004549 (PMC12503271; doi:10.1371/journal.pmed.1004549)
Supplement: S5 Table — Results are expressed as Odds Ratio (95% Confidence intervals). (DOCX) [file pmed.1004549.s008.docx]

**Table S5.** Impact of health systems strengthening on population-level coverage indicators of child, adult and maternal care (multivariable binomial regression model accounting for survey weigths, one per indicator). Results are expressed as Odds Ratio (95% Confidence intervals)

| **Indicator** | **Baseline differences between catchments** | **Changes per year in study area** | **Facility-level HSS (level of change)** | **Facility-level HSS (slope of change)** | **Program-specific HSS support^1^** | **World Bank Voucher Program** | **Covid-19 period** |
| --- | --- | --- | --- | --- | --- | --- | --- |
| **Child and adult care** |  |  |  |  |  |  |  |
| All recommended vaccines (12-23 months) | 0.68 (0.33-1.39) | 0.94 (0.82-1.08) | 0.69 (0.24-1.94) | 1.11 (0.91-1.37) | 1.79 (1.03-3.09)* | 1.35 (0.77-2.37) | 0.93 (0.51-1.68) |
| Care seeking for illness (<5 years, public provider) | 0.73 (0.44-1.2) | 1.15 (1.01-1.31)* | 4.75 (1.99-11.36)*** | 0.79 (0.64-0.97)* | 1.38 (0.79-2.42) | 0.58 (0.37-0.91)* | 0.5 (0.27-0.95)* |
| Individual care seeking for illness last 4 weeks (public provider)* | 0.91 (0.57-1.44) | 1.14 (1.01-1.27)* | 1.97 (1.17-3.3)* | 0.98 (0.9-1.08) | 1.34 (0.9-1.98) | 1.34 (0.65-2.77) | 1.39 (0.95-2.05). |
| **Maternal care** |  |  |  |  |  |  |  |
| Antenatal care (1+ visit with skilled provider) | 1.12 (0.56-2.22) | 0.96 (0.87-1.06) | 0.95 (0.39-2.31) | 1.09 (0.91-1.3) | 2.26 (1.18-4.32)* | 2.02 (1.4-2.92)*** | 1.27 (0.72-2.24) |
| Antenatal care (4+ visits with skilled provider) | 0.76 (0.42-1.37) | 1.08 (0.98-1.18) | 1 (0.38-2.59) | 1.05 (0.89-1.23) | 1.19 (0.66-2.17) | 1.37 (0.86-2.17) | 0.91 (0.57-1.45) |
| Birth delivered at public health centre | 0.71 (0.33-1.5) | 1.01 (0.88-1.16) | 1.52 (0.6-3.84) | 1.03 (0.83-1.28) | 2.03 (0.97-4.24). | 1.46 (0.87-2.45) | 0.87 (0.44-1.72) |
| Postnatal care (within 48 hours with skilled provider) | 0.72 (0.34-1.52) | 0.99 (0.86-1.14) | 1.67 (0.69-4.09) | 1.01 (0.82-1.24) | 2.05 (1.01-4.14)* | 1.26 (0.76-2.08) | 0.85 (0.44-1.66) |
| Co-coverage index (5+ interventions) | 0.74 (0.35-1.59) | 1.02 (0.91-1.15) | 1.88 (0.7-5.05) | 0.92 (0.75-1.14) | 2.58 (1.14-5.84)* | 1.1 (0.67-1.79) | 0.78 (0.45-1.36) |

*^1^ HSS support to community health program for child and adult care indicators; HSS support to maternal health program for maternal care indicators*
