# Supplementary material for: Changes in child mortality and population health following 10 years of health systems strengthening in rural Madagascar: A longitudinal cohort study
Source: PLoS Med. 2025 Oct 7;22(10):e1004549. doi: 10.1371/journal.pmed.1004549 (PMC12503271; doi:10.1371/journal.pmed.1004549)
Supplement: S6 Table — (DOCX) [file pmed.1004549.s009.docx]

**Table S6. Costing of Ifanadiana District health system per capita.**

We used a health systems perspective with top-down and bottom-up costing to determine the total per capita cost of the district health system in Ifanadiana District in 2018. We surveyed all district health facilities, including 15 health centers, one hospital, and the district office, to document infrastructure, equipment, staffing, and training using a facility assessment survey. To estimate the contribution of civil society organizations we also asked about type and amount of support received. We extracted data from district pharmacy records to determine the quantity of medicine and medical supplies procured by facilities. A review of Pivot records provided data on the NGO’s inputs. We assigned costs using financial records and estimates provided by Pivot finance and operations staff. We classified costs by level of the health system (community, health center, hospital, district) and by health system building block. We estimate the total per capita district cost by payer.

| Type of payer | Per capita input into district health system (US$) |
| --- | --- |
| **Government of Madagascar** | $18 |
| **NGO Pivot** | $35 |
| **Other partners** | $7 |
| Total | $60 |
